# Computational methods for the analysis of early-pregnancy brain ultrasonography: a systematic review

**DOI:** 10.1016/j.ebiom.2023.104466

**Published:** 2023-02-14

**Authors:** Wietske A.P. Bastiaansen, Stefan Klein, Anton H.J. Koning, Wiro J. Niessen, Régine P.M. Steegers-Theunissen, Melek Rousian

**Affiliations:** a Department of Obstetrics and Gynecology, Erasmus MC, University Medical Center, Rotterdam, the Netherlands; b Biomedical Imaging Group Rotterdam, Department of Radiology and Nuclear Medicine, Erasmus MC, University Medical Center, Rotterdam, the Netherlands; c Department of Pathology, Erasmus MC, University Medical Center, Rotterdam, the Netherlands

**Keywords:** First trimester, Embryonic and fetal brain, Ultrasonography, Computational methods

## Abstract

**Background:**

Early screening of the brain is becoming routine clinical practice. Currently, this screening is performed by manual measurements and visual analysis, which is time-consuming and prone to errors. Computational methods may support this screening. Hence, the aim of this systematic review is to gain insight into future research directions needed to bring automated early-pregnancy ultrasound analysis of the human brain to clinical practice.

**Methods:**

We searched PubMed (Medline ALL Ovid), EMBASE, Web of Science Core Collection, Cochrane Central Register of Controlled Trials, and Google Scholar, from inception until June 2022. This study is registered in PROSPERO at CRD42020189888. Studies about computational methods for the analysis of human brain ultrasonography acquired before the 20th week of pregnancy were included. The key reported attributes were: level of automation, learning-based or not, the usage of clinical routine data depicting normal and abnormal brain development, public sharing of program source code and data, and analysis of the confounding factors.

**Findings:**

Our search identified 2575 studies, of which 55 were included. 76% used an automatic method, 62% a learning-based method, 45% used clinical routine data and in addition, for 13% the data depicted abnormal development. None of the studies shared publicly the program source code and only two studies shared the data. Finally, 35% did not analyse the influence of confounding factors.

**Interpretation:**

Our review showed an interest in automatic, learning-based methods. To bring these methods to clinical practice we recommend that studies: use routine clinical data depicting both normal and abnormal development, make their dataset and program source code publicly available, and be attentive to the influence of confounding factors. Introduction of automated computational methods for early-pregnancy brain ultrasonography will save valuable time during screening, and ultimately lead to better detection, treatment and prevention of neuro-developmental disorders.

**Funding:**

The 10.13039/501100003061Erasmus MC Medical Research Advisor Committee (grant number: FB 379283).

**Research in context**

**Evidence before this study**

Early-pregnancy brain ultrasonography before 20 weeks is becoming routine clinical practice thanks to advances in ultrasound techniques, e.g. the introduction of high-frequency ultrasound probes and three-dimensional (3D) ultrasound, these advances enable early visualization of the human brain. However, monitoring growth and development and screening for abnormalities using ultrasound scans is time-consuming and prone to human errors. Automatic analysis will save time, reduce errors, and allow for multiple measurements to be taken at the same time. Hence, the aim of this systematic review was to gain insight into the future research directions needed to bring automated early-pregnancy brain ultrasonography analysis to clinical practice. In order to achieve this, we searched PubMed (Medline ALL Ovid), EMBASE, Web of Science Core Collection, Cochrane Central Register of Controlled Trials, and Google Scholar, from inception until June 2022. We included studies using ultrasound scans acquired before the 20th week of pregnancy. We included only full research papers written in the English language and no protocols, no review papers, conference abstracts, or case reports. There are several other systematic reviews focusing on computational methods for prenatal imaging. However, these reviews all focused on scans acquired during mid- and late-pregnancy and/or scans acquired with magnetic resonance imaging (MRI), and were not focused on the brain specifically.

**Added value of this study**

In this review, we created an overview of the future research directions needed to bring automated early-pregnancy brain ultrasonography analysis to clinical practice. The studies fitted in the following topics: biometry, standard plane detection, segmentation, growth models, visualization, abnormality detection and quality enhancement. The key reported attributes were: level of automation, learning-based or not, the usage of clinical routine data depicting normal and abnormal development, public sharing of program source code and data, and analysis of confounding factors. We found that the majority of the studies described the development of an automatic, learning-based method (62%). The most studied topic was biometry (40%), followed by standard plane detection (29%), segmentation (16%), growth models (7%), visualization (4%), abnormality detection (2%), and quality enhancement (2%). The majority of the studies did not use data from routine clinical care (55%). We found that none of the studies made their program source code publicly available and only two studies made the ultrasound data used publicly available. Finally, 35% of the studies did not analyse the influence of confounding factors and only 7% performed additional analyses for confounding factors beyond gestational age, image quality and body mass index.

**Implications of all the available evidence**

The findings of this systematic review show that there is an interest in automatic analysis of early-pregnancy brain ultrasonography. To bring this analysis to clinical practice we recommend that studies: use routine clinical data depicting both normal and abnormal, make their dataset and program source code publicly available, and be attentive to the influence of confounding factors. Automatic methods have the potential to drastically reduce the time needed in clinical practice for measurements of the brain and for the detection of structural abnormalities. Furthermore, automatic analysis enables the development of large-scale data-driven models. These models have the potential to provide insights into the factors influencing growth and development, which in turn may lead to early diagnosis, treatment, and prevention of neuro-development disorders.

## Introduction

The rapid development of ultrasound techniques from its introduction in 1956 has led to the implementation of prenatal two-dimensional (2D) ultrasonography in the 1970s.^1^^,^^2^ 2D ultrasonography is used for second trimester congenital anomaly screening worldwide and serves as an important baseline with regards to growth and development.^3^ Three-dimensional (3D) ultrasonography for prenatal diagnosis became available in the late 1980s, after the necessary improvement in computer technology and the introduction of transvaginal ultrasound probes.^4^ 3D ultrasonography has had a major impact on the visualization of the embryo and fetus in the first trimester. Furthermore, 3D ultrasound enables accurate biometric and volumetric measurements of structures that are hard to assess in 2D due to irregular and/or asymmetrical shapes.

During the first trimester of pregnancy, the brain is already clearly visible in ultrasonography, and its growth and structural development continue throughout pregnancy.^5^ The DOHAD paradigm (Developmental Origins of Health and Disease) states that there is a strong association between fetal growth and development and health and disease later in life.^6^ For prenatal brain development between 9 and 11 weeks gestational age associations were found with maternal age, smoking, mode of conception and folate status.^7^, ^8^, ^9^ This highlights the importance of monitoring the development of the early brain, which is reflected in the recommendation of the International Society of Ultrasound in Obstetrics and Gynecology (ISUOG) in 2021, to perform a neuro-sonographic examination in the first trimester.^10^ Since it provides us with information regarding the etiology and pathophysiology of normal and abnormal development of the human brain.

The ISUOG recommends performing the neuro-sonographic examination using a 3D trans-vaginal probe. However, when this is not feasible the examination can be performed using a 3D or 2D trans-abdominal probe. 3D ultrasonography is not always feasible due to unavailability of the equipment or lack of a trained sonographer to acquire and/or analyse the image.^10^ The recommended examination of the brain during the first trimester consists of measuring the biparietal diameter, head circumference, atrial width of the lateral ventricles and transverse cerebellar diameter. However, as pointed out by Volpe et al., by following this recommendation the majority of brain abnormalities remain undiagnosed until the second trimester.^11^ Few studies showed how to best assess the brain during the first trimester using 3D ultrasonography and how major abnormalities are characterized.^11^^,^^12^

However, monitoring growth and development and screening for abnormalities using 2D or 3D ultrasound scans is time-consuming, prone to human errors and requires specific expertise. Automatic analysis may save time, reduce errors, and allow for taking multiple measurements at the same time. Artificial Intelligence (AI) has already been shown to enable automatic analysis of images in several medical applications and can be applied to its full potential to first trimester ultrasound, as the whole embryo, thanks to its limited size, can be imaged in one dataset.^13^^,^^14^ Hence, we argue that automated analysis of ultrasonography offers an opportunity to bring early brain ultrasonography to clinical practice. The systematic review by Liu et al. showed that there is interest in developing AI for medical ultrasound analysis in different domains, but this interest is hampered by the low imaging quality of ultrasound due to noise and artifacts, and the limited amount of publicly available medical ultrasound data.^15^

Looking at related work, several systematic reviews on computational methods for prenatal imaging have been performed. Most closely related is the work by Fiorentino et al., who reviewed deep learning methods for fetal ultrasound of all gestational ages and all organs.^16^ Others reviews were focused on mid- and late-pregnancy, included only fully automatic methods, or were based on MR images.^17^, ^18^, ^19^, ^20^ However, as in clinical practice MRI is not the standard modality and mainly manual or semi-automatic methods are used to analyse the acquired images; we found these reviews too limited.

Given the potential impact of automated early-pregnancy ultrasound analysis and the lack of a systematic review covering all methods for this crucial period, we performed a systematic review covering all types of computational methods for the analysis of early-pregnancy brain ultrasonography. By creating this overview, we aim to gain insight into future research directions needed to bring automated early-pregnancy brain ultrasonography analysis to clinical practice.

## Methods

### Search strategy and selection criteria

This systematic review adheres to the PRISMA guidelines and was registered a priori at the PROSPERO registry (CRD42020189888).^21^ The specific search strategy was created together with a Health Sciences Librarian with expertise in systematic review searching. Literature search strategies were developed using medical subject headings (MeSH). We searched PubMed (Medline ALL Ovid), EMBASE, Web of Science Core Collection, Cochrane Central Register of Controlled Trials, and Google Scholar. We searched the databases from inception until June 2022. To ensure literature saturation, we scanned the reference lists of included studies, relevant reviews identified through the search and full paper proceedings of relevant international scientific conferences. Search terms used and the list of screened conference proceedings are given in Supplementary Material 1.

Literature search results were uploaded in Endnote. Two authors (WB, MR) independently screened the titles and abstracts obtained by the search against the inclusion criteria, any disagreement was resolved through discussion. Full papers were obtained for all titles that appeared to meet the inclusion criteria or when there was any uncertainty. One author (WB) screened the full papers and decided whether these met the inclusion criteria, in case of doubt the papers was discussed by WB and MR. Neither of the review authors was blinded to the journal titles or to the study authors or institutions.

Studies were selected according to the criteria outlined below. We included computational methods developed for human prenatal ultrasonography of the brain. Initially, we performed a broad search not restricted to brain ultrasonography. After title and abstract screening we obtained over 300 inclusions, which was too broad for a full text screening. Therefore, we decided to restrict ourselves to studies involving the brain only. Studies were excluded when the gestational age (GA) window of the study did not start before the 20th week of pregnancy and when the target structure of the study was not the brain or a brain structure. No restrictions on the type of data acquisition, study design, and number of subjects included in the study were applied. We included only full research papers written in the English language and no protocols, no review papers, conference abstracts, or case reports.

### Data analysis

The extracted data consisted of the following:

• year of publication;
• title;
• brain structures studied
• level of automation: manual, semi-automatic, or automatic. Here, manual refers to methods where computation can only be done after a manual action of the operator, semi-automatic refers to methods where in interaction with the operator computations are performed. For automatic methods no actions of the operator are needed.
• type of method: non-learning based, machine learning, or deep learning;
• for learning based methods the learning strategy consisting of: whether cross-validation, an external test set, and/or data augmentation was used and who provided the annotations used for learning. Additionally, for non-learning based method we report here whether an external dataset was used for evaluation.
• type of US used: 2D slices or 3D volumes;
• ultrasonography machine and probe frequency;
• number of subjects used for validation;
• GA window considered in study;
• type of data: whether or not a method used clinical routine data depicting normal and/or abnormal development;
• main outcome of the study;
• sharing of program source code and data;
• whether the software is proprietary or not: we define software as proprietary if payment is required;
• computation time;
• type of computing hardware used.

We divided all studies in the following topics: abnormality detection, biometry, growth models, segmentation, standard plane detection, quality enhancement and visualization. In abnormality detection studies the aim is to distinguish ultrasound images depicting abnormal development from images depicting normal development. Biometry studies focus on performing biometric and volumetric measurements of relevant structures within the embryonic and fetal brain. Growth model studies focus on models that describe the relationship between growth and development of the entire brain or of specific brain structures and GA. Segmentations studies focus on delineating the brain or brain structures in the ultrasound images. Standard plane detection studies focus on the detection of standard planes within the brain. Quality enhancement studies focus on improving the image quality. Finally, visualization studies focus on computational methods that visualize the embryonic or fetal brain. When studies performed tasks from multiple topics, they were classified in the category of the final topic. For example, biometric measurements are performed in standard planes, so studies that focus on biometry subsequently to standard plane detection are classified as biometry studies.

To assess the risk of bias of the studies included in this review, the ErasmusAGE quality score was used: a tool composed of five items based on previously published scoring systems that can be adapted to fit the topic of the review.^22^ Each of the five items can be allocated either zero, one or two points. The final score is the sum of the points given for each item, resulting in a total score between zero and ten. The five items are:

Q1 Study design: cross-sectional (0), longitudinal (1), intervention studies (2);
Q2 Number of subjects used for validation, the study size: ≤35 (0), 35 to 250 (1), ≥250 (2);
Q3 Description of the computational method: not reproducible based on description (0), key results are reproducible based on description (1), all results are reproducible based on description (2);
Q4 Reporting of the outcome: inadequate (0), qualitative and/or quantitative outcome reported (1), additionally: multiple raters and/or comparison to known clinical outcome (2);
Q5 Influence of confounding factors: not investigated (0), findings are analysed or adjusted for at least one of the key confounders (the influence of GA, acquisition quality and body mass index) (1), additional analysis or adjustment for confounding factors was performed (2).

Intervention studies are not applicable in this review; therefore the highest possible score is 9. The boundaries of the scoring for study size were determined by calculating the first quartile (Q1), median and third quartile (Q3) over the included full-text papers. We have chosen to evaluate only the number of subjects used for validation, rather than the total number available, since learning-based methods typically need a lot of data for development. However, the quality of the studies is generally determined by how much data is used for validation of the method, regardless of the method used. The complete quality scoring system used can be found in Supplementary Material 2.

### Statistics

No statistical tests were used. The study size in Q2 (see above) was determined as the number of subjects used for validation of the method.

### Role of funding source

The funder of the study had no role in this systematic review.

## Results

### Included studies

**Fig. 1:**
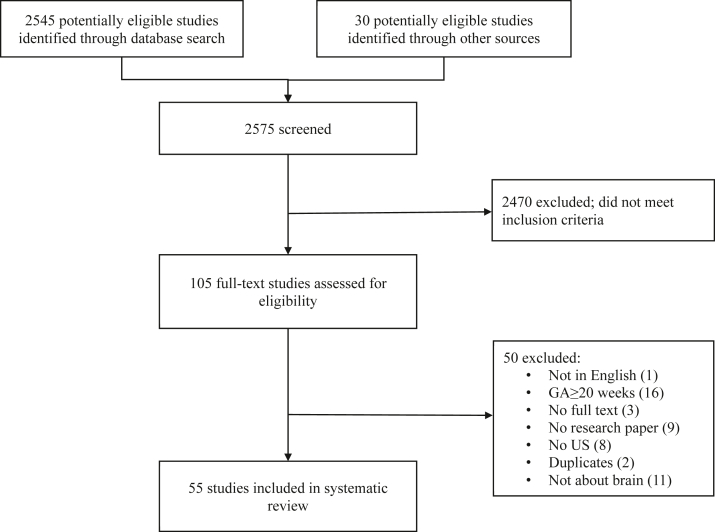
**Study selection**.

The flowchart in Fig. 1 summarizes the literature search and selection of studies. Initially, 2545 potentially eligible studies were identified through the database search, and an additional 30 potential eligible studies were identified through other sources. After title and abstract screening 105 studies remained, and the full-text was screened subsequently. We recorded the reasons for exclusion after full text screening in Fig. 1 and in Table S1 in Supplementary Material 3. After full-text screening we included 55 studies in the systematic review.

### Study characteristics

In Table 1 the main characteristics of the included studies are given. We included 22 studies on biometry, 16 studies on standard plane detection, 9 studies on segmentation, 4 studies on growth models, 1 study on abnormality detection, 1 study on quality enhancement and 2 studies on visualization. Of the included studies 4 (6%) described a manual, 9 (16%) semi-automatic, and 42 (76%) an automatic method. Regarding the type of method, 14 (25%) studies used a non-learning based method, 34 (62%) studies used a learning-based method, of which 10 (18% of the studies) used machine learning and 24 (44% of the studies) deep learning, and for 7 (13%) studies the type of method could not be identified based on the text. These seven studies used all proprietary software, of the other 10 studies using proprietary software, one was learning based. Regarding the type of data used, we found in Table 1 that for 8 (15%) it was unclear what kind of data was used, 22 (40%) studies used data acquired for research purposes, and 25 (45%) studies used clinical routine data depicting normal development. Finally, 7 (13%) studies additionally used clinical routine data depicting abnormal development and 1 study used data acquired for research purposes depicting abnormal development. None of the studies shared the program source code publicly, and two studies shared the data (23, 24). 16 (29%) studies reported the computational time, and 7 (13%) studies reported what kind of computational hardware was used.^23^, ^24^, ^25^, ^26^, ^27^, ^28^, ^29^, ^30^, ^31^, ^32^, ^33^, ^34^, ^35^, ^36^, ^37^, ^38^, ^39^, ^40^, ^41^, ^42^, ^43^ Computation time ranged from 70 micro seconds to 25 min.^32^^,^^35^ Next, we discuss the studies per topic in the following order: biometry, standard plane detection, segmentation, and together: growth models, abnormality detection, quality enhancement and visualization.

**Table 1:** General characteristics of included studies.

Topic	Author	Year	Title	Level of automation	Type of method	Computation time/hardware	Type of data	Quality score
Biometry	Araujo et al.	2014	Reference range of fetal cisterna magna volume by three-dimensional ultrasonography using the VOCAL method	SA	N^†^		R	6
Biometry	Bertucci et al.	2011	Vermian biometric parameters in the normal and abnormal fetal posterior fossa: three-dimensional sonographic study	SA	N^†^		R	4
Biometry	Birnbaum et al.	2021	Normal cavum veli interpositi at 14–17 gestational weeks: three-dimensional and Doppler transvaginal neurosonographic study	M	N^†^		R	5
Biometry	Budd et al.	2019	Confident head circumference measurement from ultrasound with real-time feedback for sonographers	A	DL		R	5
Biometry	Carneiro et al.	2008	Detection and measurement of fetal anatomies from ultrasound images using a constrained probabilistic boosting tree	A	ML	<1 s, standard dual core PC	?	6
Biometry	Cinar et al.	2020	Reference intervals and reliability of cavum septi pellucidi volume measurements by three-dimensional ultrasound between 19 and 24 weeks' gestation	SA	N^†^		C	6
Biometry	Grandjean et al.	2018	Artificial intelligence assistance for fetal head biometry: Assessment of automated measurement software	A	?^†^	<10 s	R	5
Biometry	Hata et al.	2021	Transvaginal 3-D power doppler ultrasound evaluation of the fetal brain at 10–13 weeks' gestation	SA	N^†^		R	6
Biometry	Pashaj et al.	2013	Automated ultrasonographic measurement of basic fetal growth parameters	A	ML^†^	0.1 s	C	5
Biometry	Pistorius et al.	2009	First trimester neurosonoembryology with automated follicle tracking: Preliminary findings	SA	N^†^		C∗	2
Biometry	Pluym et al.	2021	Accuracy of automated three-dimensional ultrasound imaging technique for fetal head biometry	A	?^†^	<10 s	C	6
Biometry	Rizzo et al.	2016	The feasibility of using 5D CNS software in obtaining standard fetal head measurements from volumes acquired by three-dimensional ultrasonography: Comparison with two-dimensional ultrasound	A	?^†^	54 s	C	6
Biometry	Rousian et al.	2013	First trimester brain ventricle fluid and embryonic volumes measured by three-dimensional ultrasound with the use of I-Space virtual reality	SA	N	60 s	R	6
Biometry	Ryou et al.	2019	Automated 3D ultrasound biometry planes extraction for first trimester fetal assessment	A	DL	Intel Xeon CPU at 3.50 GHz with 16.0 GB RAM	R	5
Biometry	Shehzad et al.	2007	The correlation between ultrasonic manual and automatic measurements of fetal head and abdominal circumferences	A	?^†^	CPU: 14.7s, GPU 6.9 s	R	3
Biometry	Sofka et al.	2014	Automatic detection and measurement of structures in fetal head ultrasound volumes using sequential estimation and integrated detection network (IDN)	A	DL		?	6
Biometry	Van den Heuvel et al.	2018	Automated measurement of fetal head circumference using 2D ultrasound images	A	ML		C	7
Biometry	Van den Heuvel et al.	2019	Automated fetal head detection and circumference estimation from free-hand ultrasound sweeps using deep learning in resource-limited countries	A	DL	0.00007 s for detection, 0.0005 s for HC estimation	C	6
Biometry	Verwoerd-Dikkeboom et al.	2008	Reliability of three-dimensional sonographic measurements in early pregnancy using virtual reality	M	N		R	6
Biometry	Verwoerd-Dikkeboom et al.	2010	Innovative virtual reality measurements for embryonic growth and development	M	N		R	8
Biometry	Yazdi et al.	2014	Optimal caliper placement: manual vs automated methods	A	N^†^		C	5
Biometry	Zhang et al.	2020	Direct estimation of fetal head circumference from ultrasound images based on regression CNN	A	DL		C	5
Standard plane detection	Bastiaansen et al.	2020	Towards segmentation and spatial alignment of the human embryonic brain using deep learning for atlas-based registration	A	DL		R	3
Standard plane detection	Baumgartner et al.	2016	Real-time standard scan plane detection and localisation in fetal ultrasound using fully convolutional neural networks	A	DL	39 frames per second	R	4
Standard plane detection	Burgos-Artizzu et al.	2020	Evaluation of deep convolutional neural networks for automatic classification of common maternal fetal ultrasound planes	A	DL	0.14 s	C	6
Standard plane detection	Cuingnet et al.	2013	Where is my baby? A fast fetal head auto-alignment in 3D-ultrasound	A	ML	CPU: <0.8 s	?	4
Standard plane detection	Dou et al.	2021	Agent with warm start and active termination for plane localization in 3D ultrasound	A	DL		R	4
Standard plane detection	Drukker et al.	2022	Clinical workflow of sonographers performing fetal anomaly ultrasound scans: deep learning-based analysis	A	DL		C	5
Standard plane detection	Kong et al.	2018	Automatic and efficient standard plane recognition in fetal ultrasound images via multi-scale dense networks	A	DL		C	5
Standard plane detection	Kuklisova-Murgasova et al.	2013	Registration of 3D fetal neurosonography and MRI	A	N	25 m	?	5
Standard plane detection	Namburete	2018	Fully-automated alignment of 3D fetal brain ultrasound to a canonical reference space using multi-task learning	A	DL		R	6
Standard plane detection	Rizzo et al.	2011	An algorithm based on OmniView technology to reconstruct sagittal and coronal planes of the fetal brain from volume datasets acquired by three-dimensional ultrasound	SA	?^†^	40–125 s	C∗	5
Standard plane detection	Rizzo et al.	2016	5D CNS + Software for automatically imaging axial, sagittal, and coronal planes of normal and abnormal second-trimester fetal brains	SA	?^†^	32–68 s	C∗	6
Standard plane detection	Sridar et al.	2016	Automatic identification of multiple planes of a fetal organ from 2D ultrasound images	A	DL		R	3
Standard plane detection	Welp et al.	2020	Validation of a semiautomated volumetric approach for fetal neurosonography using 5DCNS+ in clinical data from >1100 consecutive pregnancies	SA	?^†^		C	7
Standard plane detection	Yaqub et al.	2012	Automatic detection of local fetal brain structures in ultrasound images	A	ML		?	3
Standard plane detection	Yaqub et al.	2015	Guided random forests for identification of key fetal anatomy and image categorization in ultrasound scans	A	ML		C	4
Standard plane detection	Yeung et al.	2021	Learning to map 2D ultrasound images into 3D space with minimal human annotation	A	DL	0.07 s	R	6
Segmentation	Al-bander et al.	2019	Improving fetal head contour detection by object localisation with deep learning	A	DL		C	4
Segmentation	Gofer et al.	2021	Machine learning algorithms for classification of first-trimester fetal brain ultrasound images	A	ML		C∗	4
Segmentation	Gutierrez-Becker et al.	2013	Automatic segmentation of the fetal cerebellum on ultrasound volumes, using a 3D statistical shape model	A	ML	<2 s	C	5
Segmentation	Hesse et al.	2022	Subcortical segmentation of the fetal brain in 3D ultrasound using deep learning	A	DL	NVIDIA Tesla V100 (32 GB RAM)	R	7
Segmentation	Li et al.	2020	Automated measurement network for accurate segmentation and parameter modification in fetal head ultrasound images	A	DL		C	6
Segmentation	Moccia et al.	2021	Mask-R2CNN: a distance-field regression version of Mask-RCNN for fetal-head delineation in ultrasound images	A	DL	NVIDIA RTX 2080TI, with a Xeon e5 CPU and 128 GB RAM	C	6
Segmentation	Shu et al.	2022	ECAU-Net: Efficient channel attention U-Net for fetal ultrasound cerebellum segmentation	A	DL	Intel Xeon Silver 4110 2.1 GHz CPU (128 GB RAM) and two NVIDIA GTX 1080Ti GPUs (22 GB RAM)	?	3
Segmentation	Wu et al.	2017	Cascaded fully convolutional networks for automatic prenatal ultrasound image segmentation	A	DL		?	5
Segmentation	Yaqub et al.	2013	Volumetric segmentation of key fetal brain structures in 3D ultrasound	A	ML		R	3
Abnormality detection	Zhou et al.	2021	Prediction and value of ultrasound image in diagnosis of fetal central nervous system malformation under deep learning algorithm	A	DL		R∗	2
Growth model	Bihoun et al.	2020	Fetal biometry assessment with Intergrowth 21st′s and Salomon's equations in rural Burkina Faso	M	N		R	6
Growth model	Burgos- Artizzu et al.	2021	Analysis of maturation features in fetal brain ultrasound via artificial intelligence for the estimation of gestational age	A	DL		C∗	8
Growth model	Namburete et al.	2014	Predicting fetal neurodevelopmental age from ultrasound images	A	ML		C	5
Growth model	Wyburd et al.	2021	Assessment of regional cortical development through fissure based gestational age estimation in 3D fetal ultrasound	A	DL	NVIDIA GeForce RTX 2080 Ti (12 GB RAM)	R	5
Quality enhancement	Perez-Gonzalez et al.	2020	Deep learning spatial compounding from multiple fetal head ultrasound acquisitions	A	DL	3.4 s	?	4
Visualization	Pooh et al.	2016	Recent advances in 3D ultrasound, silhouette ultrasound, and sonoangiogram in fetal neurology	A	N^†^		C∗	3
Visualization	Tutschek et al.	2009	Virtual reality ultrasound imaging of the normal and abnormal fetal central nervous system	A	N^†^		C∗	4

Level of automation: M = manual, SA = semi-automatic, A = automatic. Type of method: N = not learning-based, ML = machine learning, DL = deep learning, ? = unknown, † indicates proprietary software. Type of data: R = data acquired for research, depicting normal development, C = clinical routine data, depicting normal development, ∗ indicates that method is additionally validated on imaging data depicting abnormal development. m = minutes, s = seconds.

### Biometry

**Fig. 2:**
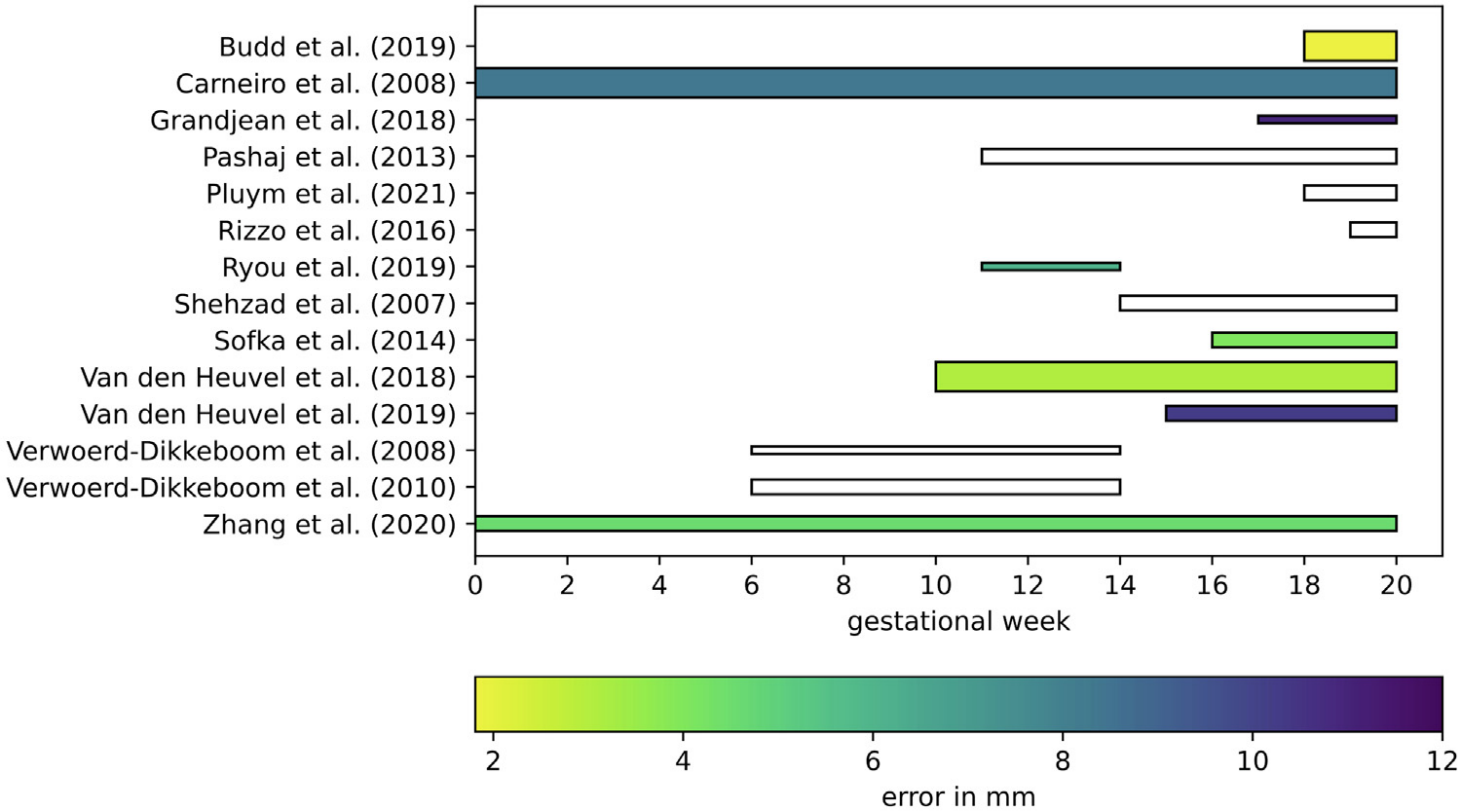
**Overview of error in the head circumference (HC) per gestational week.** The thickness of the bar indicates the number of subjects used for validation: thinnest: <35 subjects, middle: between 35 and 250 subjects, thickest: >250 subjects. A white bar indicates that no average error was reported. The error is shown up to the 20th week, since we were interested in the performance during early pregnancy.

The majority of included studies were on biometry. Detailed information about these studies is given in Table 2. The head circumference (HC) is measured most frequently: by 14 out of 22 studies.^24^, ^25^, ^26^, ^27^^,^^30^, ^31^, ^32^^,^^44^, ^45^, ^46^, ^47^, ^48^, ^49^, ^50^ In Fig. 2, for all studies measuring the HC, the GA range is given along with, if available, the mean error in millimetres (mm). Overall, the best result was achieved by Budd et al., and for the first trimester van den Heuvel et al. achieved the best results.^44^^,^^45^ Both methods used a deep learning approach, and van den Heuvel et al. made their dataset, referred to as the HC18 challenge dataset, publicly available.^44^ This dataset contains data of 335 subjects of the trans-ventricular plane. The other studies measured the cisterna magna,^51^ vermian,^52^ cavum septi pellucidi,^53^ brain volume,^54^ brain ventricles,^29^^,^^55^ the biparietal and occipitofrontal diameter,^56^ and the cavum veli interpositi, which is an interhemispheric cyst-like structure.^57^ Among the biometry studies, 11 (50%) were applied to first trimester data (<14 weeks GA).^26^^,^^29^^,^^30^^,^^44^^,^^48^, ^49^, ^50^^,^^54^^,^^55^^,^^57^^,^^58^

**Table 2:** Detailed information on the biometry studies.

Author	Year	Type of method	Brain structure	GA	Description of method	Learning strategy (if applicable)	US machine, US Probe, 2D/3D	Number of subjects	Outcome
Araujo et al.	2014	N	CM	17–29	4D view^†^ software, VOCAL function.		Voluson 730^†^ ? 3D	224	ICC: 0.92
Bertucci et al.	2011	N	Vermian perimeter, cross-sectional area, and super inferior diameter	18–35	4Dview^†^ software.	External test set	Voluson 730 or E8^†^, 4–8 MHz TAb or 5–9 MHz TVa, 3D	12	Significantly smaller cross-sectional area (18-19w), and perimeter (28-29w) in abnormal cases
Birnbaum et al.	2021	N	Cavum veli interpositi, an interhemispheric cyst-like structure	14–17	4Dview^†^ software.		Voluson E6, E8 or E10^†,^ 5–9 MHz TVa, 3D	87	TCD: 13.1–18.4 mm Cavum veli interpositi: 0.3–0.8 mm Detection: 45%
Budd et al.	2019	DL	HC	18–22	A U-net was used for segmentation, followed by ellipse fitting to determine the HC. Using Monte Carlo drop-out an ensemble of segmentation was obtained; cases with high variance for the HC estimation were rejected.	Data-augmentation: flipping, rotation, Annotations: expert sonographers Strategy: no cross-validation, external test set	?, ?, 2D	540	Error = 1.81 mm
Carneiro et al.	2008	ML	BPD, HC	All	Feature extraction of image regions that were segmented by a constrained probabilistic tree classifier. From the segmentation the measurements were derived.	Data-augmentation: none Annotations: 15 expert sonographers Strategy: no cross-validation, 3 external test sets	?, ? 2D	1760	Error: BPD = 2.73 mm HC = 8.34 mm
Cinar et al.	2020	N	Cavum septi pellucidi	19–24	4D view^†^ software, VOCAL software function.		Voluson E6^†^, 2–7 MHz TAb, 3D	99	ICC: Intermediate – experienced: 0.78 Novice – experienced: 0.50 Novice – intermediate: 0.57
Grandjean et al.	2018	?	BPD, HC	17–29	Smartplanes^‡^ software.		Resona 7^‡^, 5–8 MHz, 3D	30	Error: BPD = 4 mm, HC = 11 mm
Hata et al.	2012	N	BV	10–13	4D view^†^ software, VOCAL software function.		Voluson E8^†^, 3.7–17.5 MHz TVa, 3D	36	ICC: 0.991
Pashaj et al.	2013	ML	BPD, OFD, HC	11–40	Syngo auto OB^§^ software.	Annotations: not mentioned	? ^§^, 2.5–6 MHz, 2D	83	Success rate: BPD = 79.89%, OFD = 81.80% HC = 85.97%
Pistorius et al.	2009	N	Ventricles of telencephalon, diencephalon, mesencephalon and rhombencephalon	8–9	4D view^†^ software.		Voluson E8^†^, 6–12 MHz TVa, 3D	6	Success rate: 66% for all ventricles
Pluym et al.	2021	?	BPD, HC, TCD, CM, LV	18–22	SonoCNS^†^ Fetal Brain software.		Voluson E10^†^, 2–8 MHz Tab, 2D	143	ICC: BPD = 0.81 HC = 0.88 TCD = 0.50 CM = 0.23 LV = 0.26
Rizzo et al.	2016	?	BPD, HC, TCD, CM	19–22	5D CNS^¶^ software.		WS80A Elite^¶^, 1–8 MHz TAb, 3D	120	ICC: BPD = 0.974 HC = 0.995 TCD = 0.994 CM = 0.990
Rousian et al.	2013	N	Brain ventricle fluid volume	6–12	BARCO I-Space VR system, V-Scope volume rendering software.		Voluson E8^†^, 4.5–11.9 MHz TVa, 3D	112∗	Success rate: 38%
Ryou et al.	2019	DL	HC	11–14	2D slices of the image were used as input for a multi-task FCNN which outputs the segmentation of head, embryo and limbs and classification of the plane. These steps were repeated for slices taken from all three views (coronal, sagittal and axial). To obtain the HC, ellipse fitting was used.	Data-augmentation: none Annotations: checked by clinicians Strategy: no cross-validation, external test set	HD9∗∗, V7-3, 2D	21	Error = 6.03 mm
Shehzad et al.	2007	?	HC	14–38	Automatic ellipsoid mode software^††^.		EcoCee and Power Vision^††^, 3.0–4.2 MHz, 2D	72	Correlation = 0.9999, Mean: significantly different
Sofka et al.	2014	DL	HC, BPD, OFD, LV, CM, CER	16–35	A sequential estimation and integrated detection network was used, which employs the spatial relationship between different measurements. This was used to guide training of the network to detect the HC.	Data-augmentation: flipping. Annotations: 1 experienced sonographer Strategy: no cross-validation, external test set	Antares and S2000^§^, ?, 3D	107	error: CER = 1.37 mm CM = 0.87 mm LV = 1.01 mm OFD = 2.31 mm BPD = 0.94 mm HC = 4.06 mm
Van den Heuvel et al.	2018	ML	HC	10–40	Haar-like features were extracted from the image and were used as input for a Random Forest to detect the fetal skull. The HC was extracted using the Hough transform, dynamic programming and ellipse fitting.	Data-augmentation: none Annotations: during acquisition, trained medical researcher Strategy: three-fold cross-validation, external test set	Voluson E8 or 730^†^, ?, 2D	335	Error: first trim. = 3.1 mm second trim. = 2.5 mm third trim. = 4.8 mm
Van den Heuvel et al.	2019	DL	HC	15–40	A two-step approach for minimum computational resource circumstances used the well-known VGG-net for detection of the head and U-net for HC estimation via segmentation	Data-augmentation: flipping. Annotations: 1 experienced sonographer Strategy: no cross-validation, external test set	SonoAce R3^¶^, ?, 2D	39	Error = 10.3 mm
Verwoerd-Dikkeboom et al.	2008	N	HC, BPD	6–14	I-space, a virtual reality system that uses a virtual pointer to measure length.		Voluson 730^†^, ?, 3D	28∗	ICC: BPD = 0.998 HC = 0.997
Verwoerd-Dikkeboom et al.	2010	N	BPD, HC, OFD	6–14	I-space, a virtual reality system that uses a virtual pointer to measure length.		Voluson 730^†^, ?, 3D	125∗	Success rate: BPD = 96.8% OFD = 96.8% HC = 96.8%
Yazdi et al.	2014	DL	BPD, OFD	19–25	SonoBiometry^†^ software.	Annotations: two experts, one resident and two students	Voluson E8^†^, ?, 2D	95	Error: BPD = −0.17 mm OFD = −0.06 mm
Zhang et al.	2020	DL	HC	0–40	Regression of features extracted by a CNN to predict the HC. The fetal head is not segmented explicitly.	Data-augmentation: flipping, translation and rotation Annotations: during acquisition, trained medical researcher Strategy: 5-fold cross-validation, external test set	Voluson E8 or 730^†^, ?, 2D	199	Error = 4.52 mm

Legend to brain structures: BPD = biparietal diameter, CER = cerebellum, CM = cisterna magna, HC = head circumference, LV = lateral ventricles, OFD = occipitofrontal dimeter, TCD = transverse cerebellar diameter. Legend to description of method: CNN = convolutional neural network, FCNN = fully convolutional neural network; a brief explanation can be found in Supplementary Material 4. 2D = two-dimensional, 3D = three dimensional, a ∗ indicates longitudinal data. ICC = Intraclass Correlation Coefficient. †GE Medical Systems, Zipf, Austria, ^‡^Mindray, Shenzen, China, §Siemens, USA, ^¶^Samsung Medison, Korea, ∗∗Philips, Bothell, WA 98021, USA, ^††^Toshiba, Japan. TAb = transabdominal, TVa = transvaginal.

Biometric measurements are performed in their corresponding standard plane. Most studies used 2D slices from the 3D volume or 2D images that were manually acquired and annotated by experienced sonographers. Only Grandjean et al., Ryou et al., and Sofka et al. used the entire 3D volume as input. Grandjean et al. used proprietary software, and both Ryou et al. and Sofka et al. used an additional deep learning approach to first detect the required standard plane for each measurement.^25^^,^^30^^,^^47^ Ryou et al. first classified each slice as containing the head or abdomen, which was correct in 98.9% of the cases, and subsequently used regression to find the biometry plane.^30^ Sofka et al. first detected landmarks to find the required standard plane, and reported an average landmark detection error of less than 2 mm.^47^

### Standard plane detection

**Fig. 3:**
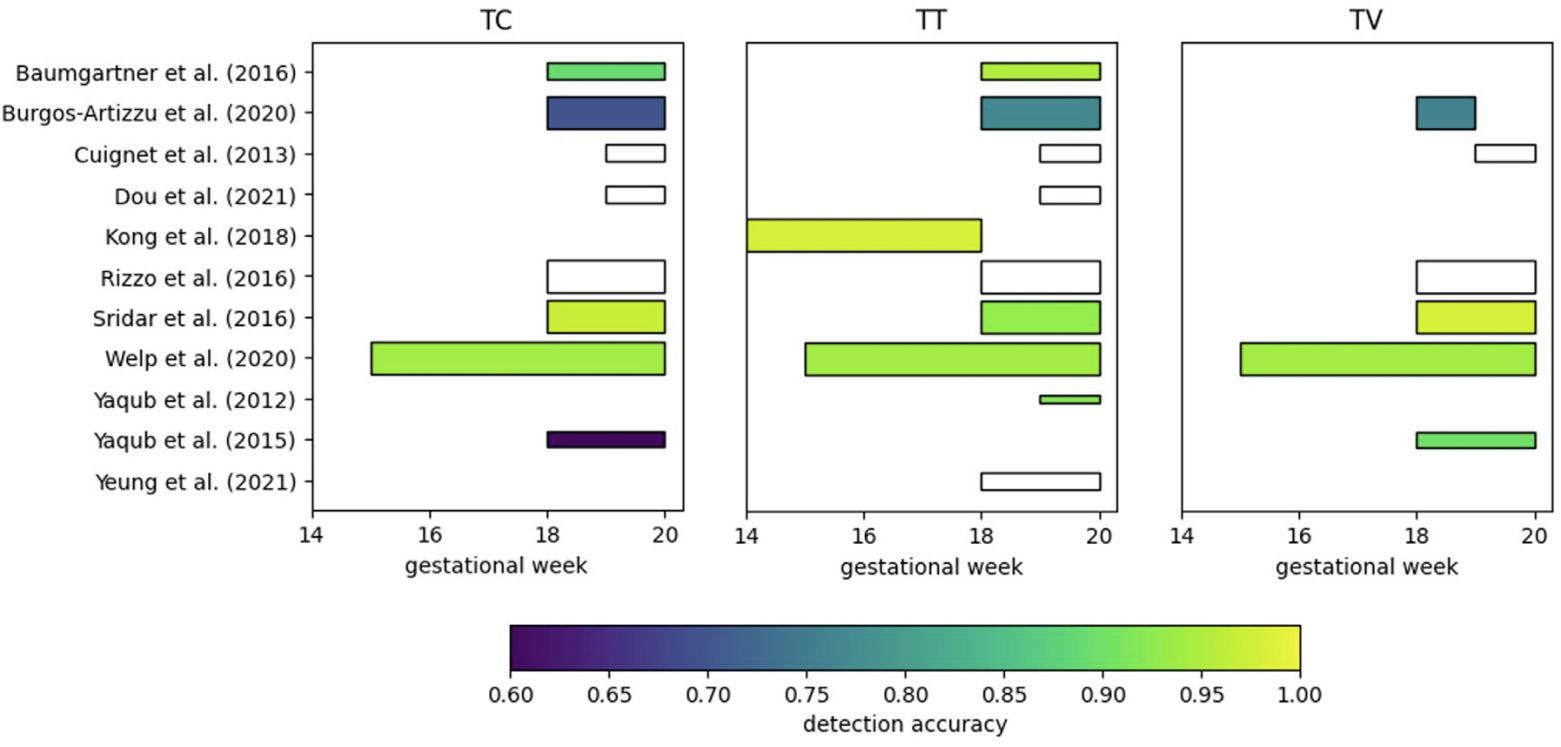
**Overview of detection accuracy of the trans-cerebellar plane (TC), trans-thalamic plane (TT) and trans-ventricular plane (TV) per week gestational age**. The thickness of the bar indicates the number of subjects used for validation: thinnest: <35 subjects, middle: between 35 and 250 subjects, thickest: >250 subjects. A white bar with a black edge indicates that no accuracy was reported. The accuracy is shown up to the 20th week, since we were interested in the performance during early pregnancy.

For standard plane detection the detailed information is given in Table 3. All 16 studies used automatic methods. In 11 out of the 16 studies the trans-cerebellar (TV), trans-thalamic (TT) and/or trans-ventricular (TV) plane was detected. Fig. 3 gives an overview of the detection accuracy and the GA range.^23^^,^^28^^,^^33^^,^^34^^,^^37^^,^^59^, ^60^, ^61^, ^62^, ^63^, ^64^ The best results for the TT plane were achieved by Kong et al. and the best results for the TC and TV plane were achieved by Sridar et al.^60^^,^^61^ Burgos-Artizzu et al. made their dataset publicly available, which consists of data of 1792 patients, and contains besides the TC, TT and TV plane, the abdominal, femur and thorax standard planes.^23^ The other studies detected the brain,^35^^,^^65^, ^66^, ^67^ or other standard planes.^28^^,^^37^Only the study by Bastiaansen et al. was applied to first trimester data (<14 weeks GA).^65^

**Table 3:** Detailed information on the standard plane detection studies.

Author	Year	Type of method	Brain structure	GA	Description of method	Learning strategy (if applicable)	US machine, US Probe, 2D/3D	Number of subjects	Outcome
Bastiaansen et al.	2020	DL	Brain	9	A CNN was trained to register an image to an atlas. The atlas consisted of an ultrasound image put in a pre-defined orientation and the brain was segmented. By learning the correspondence between image and atlas the fetal brain was detected.	Data-augmentation: flipping, rotation, translation, zooming, Annotations: not mentioned Strategy: no cross-validation, external test set	Voluson E8^†^, 4.5–11.9 MHz TVa, 3D	30∗	Success rate = 27%
Baumgartner et al.	2016	DL	TC, TT	18–22	A FCNN was trained to predict which standard plane is shown. A one-to-one correspondence between each feature map in the network and prediction was enforced. From this correspondence, a confidence map was derived. The prediction with the highest confidence was outputted.	Data-augmentation: flipping, rotation, translation Annotations: team of expert sonographers Strategy: no cross-validation, external test set	Voluson E8^†^, ?, 2D	201	Accuracy: TC = 0.89 TT = 0.95
Burgos-Artizzu et al.	2020	DL	TC, TT, TV	18–40	Three well-known classification architectures (VGG-net, ResNet and DenseNet) were compared for common hyperparameter choices to detect brain, abdomen, cervix, femur and thorax standard planes. The most successful architecture was DenseNet, this was subsequently trained to detect the TC, TT and TV within the brain class.	Data-augmentation: flipping, cropping, translation and rotation, Annotations: senior maternal–fetal specialist Strategy: no cross-validation, external test set	Voluson E6, S8, S10^†^ and Aloka 3–7.5 MHz (TAb), 2–10 MhZ (TVa) 2D	536	Accuracy: TC = 0.70 TT = 0.77 TV = 0.76
Cuingnet et al.	2013	ML	TC, TT, TV	19–24	First, the skull was detected using a shape model and template deformation. Next, the midsagittal plane (MSP) was detected using the Hough transform and eye orbits were detected using a Random Forest trained on geometric information and image features. From the positions of the skull, MSP and the eye orbits the standard planes can be derived.	Data-augmentation: none Annotations: not mentioned Strategy: two-fold cross-validation, no external test set	?, ?, 3D	78	Median error: TC = 5.8 mm TT = 5.1 mm TV = 5.3 mm
Dou et al.	2021	DL	TC, TT	19–31	A reinforcement learning approach is used. The network was initialized with a so-called landmark-aware alignment module, where anatomical landmarks were detected and aligned with a plane-specific atlas.	Data-augmentation: none Annotations: expert sonographers with 5 years of experience Strategy: no cross-validation, external test set	DC-9^‡^, ?, 3D	100	Error: TC = 3.40 mm TT = 2.66 mm
Drukker et al.	2022	DL	Brain, face in sagittal plane, face in coronal plane	19–21	A deep spatio-temporal model was trained to label short scan clips of standard plane acquisition. This gave insight in the number of correctly detected standard planes and the order of acquisition.	Data-augmentation: none Annotations: four experts Strategy: no cross-validation, external test set	Voluson E8, ?, 2D	496	Accuracy: Brain: 0.97 Sagittal face: 0.79 Coronal face: 0.86
Kong et al.	2018	DL	TT	14–18	A multi-scale dense NN was used, consisting of a cascade of neural networks, all operating on a different spatial resolution of the input- and output image, to learn both global and local information.	Data-augmentation: none Annotations: during acquisition Strategy: 5-fold cross-validation, external test set	?, ?, 2D	5700	Accuracy = 0.98
Kuklisova-Murgasova et al.	2013	N	Brain, landmarks for CP, VC, CH, CSP	18–22, 28	The brain was aligned by finding the location of the landmarks for the CP, VC, CH and CSP. This was done via block-matching of ultrasounds with so-called pseudo ultrasound. These pseudo ultrasound images were derived from MRI images with known positions of the landmarks. The brain was detected using the alignment of the pseudo ultrasound and clinical ultrasound.	External test set	HD 9 or IU22^§^, ?, 3D	34	Dice, error: CP = 0.58, 2.38 mm VC = 0.46, 2.42 mm CH = 0.57, 1.87 mm CSP = 0.43, 2.77 mm
Namburete et al.	2018	DL	Brain	18–34	A multi-task FCNN learning approach was used. The transformation to align the brain was derived from the orientation of individual 2D slices, the position of the eye orbits, and the segmented brain.	Data-augmentation: none Annotations: not mentioned Strategy: no cross-validation, external test set	HD 9^§^, 2.5 MHz, 3D	140	Dice = 0.82
Rizzo et al.	2011	?	TCaudc, TFc, TTc, TCc	18–24	OmniView^†^ software.		Voluson 8^†^, 4–8 MHz TAb, 3D	105	Cohen's kappa: Tcaudc = 0.89, TFc = 0.93 TTc = 0.92 TCc = 0.93
Rizzo et al.	2016	?	TC, TCaudc, TCc, TFc, TT, TTc, TV	18–24	5DCNS+^¶^ software.		WS80A Elite^¶^, 1–8 MHz TAb, 3D	205	Cohen's kappa: TC = 0.97 Tcaudc = 0.89 TCc = 0.94 TFc = 0.90 TT = 0.98 TTc = 0.92 TV = 0.96
Sridar et al.	2016	DL	TC, TT, TV	18–20	A pre-trained CNN was used to extract features. Using these features a SVM was trained to classify the standard planes.	Data-augmentation: cropping, flipping Annotations: medical imaging researcher under clinical supervision Strategy: no cross-validation, external test set	Voluson E8^†^, ?, 2D	85	Accuracy: TV = 0.98 TT = 0.93 TC = 0.97
Welp et al.	2020	?	TV, TT, TC, TFc, Tcaudc, TTc, TCc	15–36	5DCNS+¶ software.		WS80A Elite¶, 1–8 MHz TAb, 3D	1019	Success rate: 8/9 planes = 98%, 9/9 planes = 94%
Yaqub et al.	2012	ML	Detection of the TT by detection of: CP, VC, CSP, CER	19–24	A Random Forest was used to detect the CP, VC, CSP and CER, from extracted Haar, cuboid, binary and unary features. From the detected position the TT plane was derived.	Data-augmentation: none Annotations: experienced sonographer Strategy: 10-fold cross-validation, external test set	?, ?, 3D	30	Accuracy: CP = 0.93 VC = 0.91 CSP = 0.92 CER = 0.92
Yaqub et al.	2015	ML	TC, TV	18–22	A Random Forest was trained guided by features that indicate the relevance of structures in the image. These features were calculated by comparing the image to a template.	Data-augmentation: Cropping, flipping Annotations: 14 qualified sonographers Strategy: 10-fold cross-validation, no external test set	Voluson E8^†^, ?, 2D	200	Accuracy: TV = 0.90 TC = 0.60
Yeung et al.	2021	DL	TT	18–22	A regression CNN was trained that consists of four sequential modules: a feature extracting CNN, a comparison module for the extracted features, an attention mechanism to weigh the contribution of each comparison, and a prediction module to predict the position in 3D space.	Data-augmentation: rotation Annotations: not mentioned Strategy: 10-fold cross-validation, external test set	HD 9^§^, 25 MHz, 3D	189	Error: 11.4 voxels

Legend to brain structures: CER = cerebellum, CH = cerebellar hemisphere, CP = choroid plexus, CSP = cavum septi pellucidi, TC = transcerebellar plane, TCc = coronal transcerebellar plane, TCaudc = coronal transcaudate plane, TFc = coronal transfrontal plane, TT = transthalamic plane, TTc = coronal transthalamic plane, TV = transventricular plane, VC = posterior ventricular cavity. Legend to description of method: CNN = convolutional neural network, FCNN = fully convolutional neural network, NN = neural network, SVM = support vector machine; a brief explanation can be found in Supplementary Material 4. 2D = two-dimensional, 3D = three-dimensional, a ∗ indicates longitudinal data. ^†^GE Medical Systems, Zipf, Austria, ^‡^Mindray, Shenzen, China, §Philips, Bothell, WA 98021, USA, ^¶^Samsung Medison, Korea, ∗∗Aloka CO., LTD. TAb = transabdominal.

### Segmentation

**Fig. 4:**
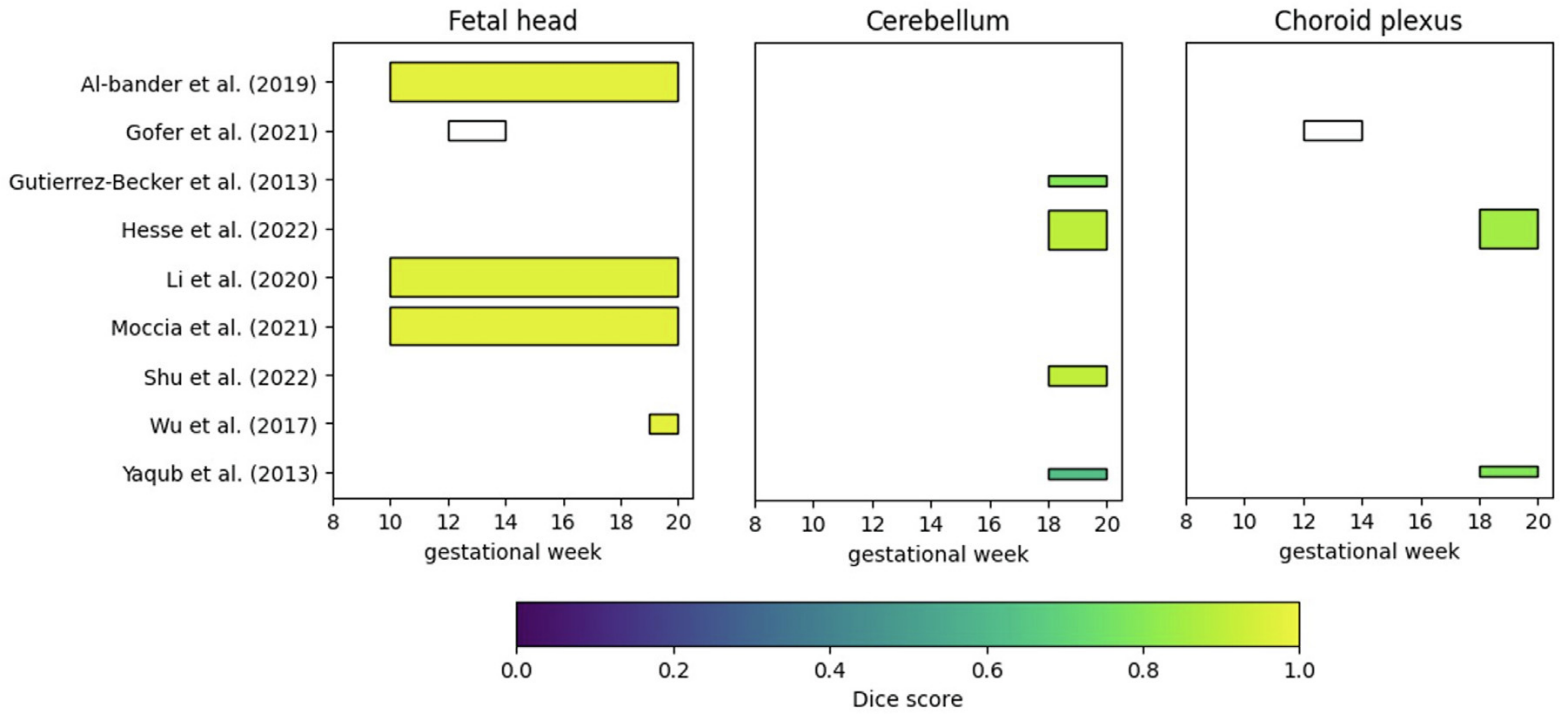
**Overview of the Dice score for segmentation of the fetal head in the trans-ventricular (TV) plane, cerebellum (CER) and choroid plexus (CP) per week gestational age**. The thickness of the bar indicates the number of subjects used for validation: thinnest: <35 subjects, middle: between 35 and 250 subjects, thickest: >250 subjects. A white bar with a black edge indicates that no Dice score was reported. The Dice score is shown up to the 20th week, since we were interested in the performance during early pregnancy.

Table 4 gives the detailed information about the segmentation studies. The fetal head in the TV plane was segmented by five studies.^38^^,^^40^^,^^68^, ^69^, ^70^ Three of those used the aforementioned HC18 challenge dataset.^40^^,^^44^^,^^68^^,^^69^ Furthermore, four studies segmented the cerebellum,^39^^,^^41^^,^^71^^,^^72^ and three studies segmented the choroid plexus.^38^^,^^39^^,^^72^ All these studies used a learning-based method, and four studies used data acquired in the first trimester (<14 weeks).^38^^,^^40^^,^^68^^,^^69^ Fig. 4 gives an overview of the Dice score and GA range for all studies.

**Table 4:** Detailed information on the segmentation studies.

Author	Year	Type of method	Brain structure	Gestational age	Description of method	Learning strategy (if applicable)	US machine, US Probe, 2D/3D	Number of subjects	Outcome
Al-Bander et al.	2019	DL	Fetal head	10–40	A FCNN was trained for segmentation and refined using ellipse fitting.	Data-augmentation: cropping, rotation, zooming Annotations: during acquisition, trained medical researcher Strategy: no cross-validation, external test set	Voluson E8 or 730^†^, ?, 2D	335	Dice = 0.98
Gofer et al.	2021	ML	Fetal head, CP	12–14	Two segmentation algorithms were compared: 1) statistical region merging, which uses image intensities, and 2) trainable Weka segmentation, which is based on an ensemble of machine learning algorithms. Trainable weka segmentation performed best.	Data-augmentation: none Annotations: two obstetricians with a subspecialty in fetal imaging Strategy: k-fold cross-validation, no external test set	Voluson E10^†^, 6–12 MHz TVa, 3D	56	Mean percentage error: 1.71%
Gutierrez-Becker et al.	2013	ML	CER	18–24	A point distribution model was used. This model is a special case of statistical shape model. Thirty points were used to segment the cerebellum.	Data-augmentation: none Annotations: expert fetal medicine specialist Strategy: 20-fold cross-validation, no external test set	Voluson 730^†^, 4–8 MHz, 3D	20	Dice = 0.80
Hesse et al.	2022	DL	CP, LV, CSP, CER	18–26	A 3D U-net for segmentation was trained using only 9 fully annotated volumes, combined with many weakly labeled volumes obtained from atlas-based segmentations.	Data-augmentation: none Annotations: two experienced sonographers Strategy: no cross-validation, external test set	Philips HD 9^‡^, TAb, 3D	278	CP = 0.85 LV = 0.85 CSP = 0.78 CER = 0.90
Li et al.	2020	DL	Fetal head	10–40	A FCNN was trained, combined with ellipse fitting for the final segmentation. Simultaneously, fetal head measurements were performed with a special regression branch to regularize the segmentation result.	Data-augmentation: Brightness, contrast, sharpness, Gaussian blur, flipping Annotations: during acquisition, trained medical researcher Strategy: no cross-validation, external test set	Voluson E8 or 730^†^, ?, 2D	335	Dice = 0.97
Moccia et al.	2021	DL	Fetal head	10–40	A CNN was trained to predict the HC distance field, bounding box, and segmentation of the fetal head. The CNN is based on a recurrent neural network, which is a specific type of architecture designed to propagate information across images.	Data-augmentation: scaling, translation, rotation and shearing Annotations: during acquisition, trained medical researcher Strategy: no cross-validation, external test set	Voluson E8 or 730^†^, ?, 2D	335	Dice = 0.98
Shu et al.	2022	DL	CER	18–26	A U-net was combined with an adaptive soft attention module for segmentation. This attention module makes use of convolutional layers instead of fully connected layers.	Data-augmentation: flipping, Gaussian blur Annotations: radiologist of the ultrasound department Strategy: no cross-validation, external test set	Voluson E10^†^, 2.5–7 MHz Tab, 2D	192	Dice = 0.91
Wu et al.	2017	DL	Fetal head	19–40	A cascaded FCNN was trained, which consists of multiple so-called levels of a FCNN. Every level uses information learned in the previous level.	Data-augmentation: none Annotations: during acquisition, trained medical researcher Strategy: no cross-validation, external test set	?, ?, 2D	236	Dice = 0.98
Yaqub et al.	2013	ML	CP, CSP, CER, VC	18–26	A Random Forest was trained for segmentation. Distance features for the skull, the center of the head, and eye orbits were used besides classical image features.	Data-augmentation: none Annotations: experienced clinician Strategy: no cross-validation, external test set	iU22^‡,^ ?, 3D	20	Dice: CP = 0.79 CSP = 0.74 CER = 0.63 VC = 0.82

Legend to brain structures: CER = cerebellum, CP = choroid plexus, CSP = cavum septi pellucidi, LV = lateral ventricles, VC = posterior ventricular cavity. Legend to description of method: CNN = convolutional neural network, FCNN = fully convolutional neural network; a brief explanation can be found in Supplementary Material 4. 2D = two-dimensional, 3D = three-dimensional, a ∗ indicates longitudinal data. ^†^GE Medical Systems, Zipf, Austria, ^‡^Philips, Bothell, WA 98021, USA.

### Growth models, abnormality detection, quality enhancement and visualization

Table 5 gives the details of the studies about growth models, abnormality detection, quality enhancement and visualization. Four studies present a growth model.^42^^,^^73^, ^74^, ^75^ Bihoun et al. compared the growth curves for a population from rural Burkina Faso, using the Salomon equation and the Intergrowth 21-st growth curves.^73^ Burgos-Artizzu et al. estimated the GA based on the TT plane using a deep learning approach.^74^ In a comparable study, Namburete et al. predicted the GA from the appearance of the Silvian Fissure using a machine learning method.^75^ Finally, Wyburd et al. estimated the GA based on the Sylvian fissure, parieto-occipital fissure and calcarine sulcus.^42^ Wyburd et al. had the lowest errors of 3.4 days, using the Sylvian fissure. For abnormality detection, Zhou et al. developed a deep learning-based method based on 2D brain slices, with an accuracy of 65%.^76^ Perez-Gonzalez et al. developed an automatic method for quality enhancement, by merging several partially occluded ultrasound images of the same object.^43^ Finally, the studies of Pooh et al. and Tutscheck et al. focused on visualization of the fetal brain during the first trimester.^77^^,^^78^

**Table 5:** Detailed information on the growth model, quality enhancement and visualization studies.

Topic	Author	Year	Type of method	Brain structure	Gestational age	Description of method	Learning strategy (if applicable)	US machine, US Probe, 2D/3D	Number of subjects	Outcome
Abnormality detection	Zhou et al.	2021	DL	Brain	17–32	A CNN for classification was combined with the Java fuzzy cognitive maps algorithm to filter the found features before final classification.	Data-augmentation: none Annotations: diagnosed based on the pathological results of the fetus Strategy: not mentioned	Voluson E8^§^, ?, 2D	?	Accuracy: Week 17–19: 0.64
Growth model	Bihoun et al.	2020	N	BPD, HC	16–36	Comparison of the resulting growth curve based on Salomon equation and the Intergrowth 21-st growth curves was performed, for a population from rural Burkina Faso.		FFsonic UF-4100^†^, 3.5–5.0 MHz TAb, 2D	276	Error = - 0.01 mm for HC
Growth model	Burgos-Artizzu et al.	2021	DL	TT	16–42	A CNN, pre-trained to detect key brain structures, was trained to estimate the gestational age from the brain image. Within the architecture of the CNN, regular convolutions were replaced by a series of slightly altered coordinated convolution layers, which incorporated image resolution into the computation.	Data-augmentation: none Annotations: GA was determined by CRL measurements on first-trimester ultrasound Strategy: no cross-validation, external test set	Voluson E6, S8 and S10^§^, and Aloka^¶^, 3–7 MHz Tab, 2D	598	Error: 14.2 days
Growth model	Namburete et al.	2014	ML	Silvian fissure	18–27	A Regression Forest was trained on image features extracted from the Silvian fissure to predict the GA of the given image.	Data-augmentation: none Annotations: combination of first day last menstural period (LMP) and first trimester US measurements Strategy: 12-fold cross-validation, external test set	HD9^‡^, 2–5 Mhz, 3D	32	Error: left hemisphere = 6.11 days right hemisphere = 6.66 days
Growth model	Wyburd et al.	2021	DL	Sylvian fissure, parieto-occipital fissure, calcarine sulcus	19–30	The 3D VGG-Net and 3D ResNet architectures were compared to predict the GA from the different structures. Furthermore, attention maps for GA prediction were studied for the different structures.	Data-augmentation: none Annotations: combination of first day last menstural period (LMP) and first trimester US measurements Strategy: 12-fold cross-validation, external test set	?, ?, 3D	811	Error: Sylvian fissure: 3.4 days Parieto-occipital fissure: 4.9 days Calcarine sulcus: 5.0 days
Quality enhancement	Perez-Gonzalez et al.	2020	DL	Brain	14–27	Several partially occluded ultrasound images of the same object were merged using a pipeline of CNNs. Two CNNs were used to segment the fetal skull, one was used to register the fetal brain to a common reference space, and the final CNN was used to merge different acquisitions together by learning how to weigh their influence on the resulting image.	Data-augmentation: none Annotations: expert obstetrician Strategy: cross-validation, no external test set	?, 8–20 MHz, 3D	18	Increase image sharpness: 34.9%
Visualization	Pooh et al.	2016	N	Brain	8–31	HDlive^§^ software was used to visualize the cerebral vascular structure.		Voluson E10^§^, 6–12 MHz TVa, 3D		
Visualization	Tutschek et al.	2009	N	Atrium, LV, corpus callosum, CER, cerebellar vermis, CM, CP, CSP, falx cerebri, frontal horns, interhemispheric fissure, occipital horns, thalami, temporal horns	Late first trimester to mid-trimester	4Dview^§^ software.		Voluson 730 or E8^§^, ?, 3D	22	
Visualization	Tutschek et al.	2009	N	Atrium, LV, corpus callosum, CER, cerebellar vermis, CM, CP, CSP, falx cerebri, frontal horns, interhemispheric fissure, occipital horns, thalami, temporal horns	Late first trimester to mid-trimester	4Dview^§^ software.		Voluson 730 or E8^§^, ?, 3D	22	

Legend to brain structures: BPD = biparietal diameter, CER = cerebellum, CM = cistera magna, CP = choroid plexus, CSP = cavum septi pelllucidi, LV = lateral ventricles, TT = trans-thalamic plane. Legend to description of method: CNN = convolutional neural network, VGG-net, ResNet = widely used network architectures; a brief explanation can be found in Supplementary Material 4. 2D = two dimensional, 3D = three-dimensional, a ∗ indicates longitudinal data. ^†^Fukunda Denshi, ^‡^Philips, Bothell, WA 98021, USA, ^§^GE Medical Systems, Zipf, Austria, ^¶^Aloka Co, Ltd, Tokyo, Japan.

### 2D versus 3D ultrasonography

We found 23 (43%) studies using 2D ultrasonography and 31 (57%) studies using 3D ultrasonography. The studies using 2D data were mainly using automatic methods (96%), and made use of deep learning (65%). For the studies using 3D data there was no main type of method used: there were 19 (61%) automatic, 9 (29%) semi-automatic and 3 (10%) manual methods, of which 8 (26%) used deep learning, 6 (19%) machine learning, and 12 (39%) were not learning-based. Only 9 (29%) of the studies using 3D ultrasonography mentioned that the ultrasound was acquired trans-vaginally, as recommended by the ISUOG. For the most studied topics, biometry, standard plane detection and segmentation, the included studies used both 2D and 3D ultrasonography, for biometry in 45% of the studies 2D ultrasonography was used and in 55% of the studies 3D ultrasonography was used, for standard plane detection this was 33% versus 66%, and for segmentation 55% versus 45%.

### Learning strategy

For the 34 studies using a learning-based approach we reported the usage of data-augmentation, an external test, and cross-validation. Furthermore, we reported who provided the annotations used for evaluation. Of the 34 studies, 14 (41%) used data-augmentation.^23^^,^^32^^,^^33^^,^^37^^,^^40^^,^^41^^,^^45^^,^^47^^,^^50^^,^^61^^,^^64^^,^^65^^,^^68^^,^^69^ Flipping and rotation were the most used in 71% and respectively 57% of the cases. Five (15%) studies used only cross-validation, 19 (56%) studies used only an external test set, 7 (20%) studies used both, and for three (9%) studies this was not mentioned. Regarding the annotations, they were mainly provided by a single clinical expert (44%), multiple experts (21%), or trained researchers under supervision of a clinical expert (24%).

### Risk of bias

For all included studies we found quality scores between 2 and 8, with a median of 5. The total quality score for each study can be found in Table 1, and the scores given per item can be found in Table S2 in Supplementary Material 5. There were only four (7%) studies using longitudinal data^29^^,^^48^^,^^49^^,^^74^ and only two of the studies gave an unreproducible description of their method.^67^^,^^76^ Furthermore, 27 (49%) studies had, besides qualitative and/or quantitative reporting of outcome, additionally multiple raters or compared their result to known clinical outcomes. Regarding analysing the influence of confounders, 19 (35%) studies did not adjust or analyse the influence of at least one of the key confounders (GA, acquisition quality or body mass index) and only four (7%) studies performed an analysis to identify or adjust for additional confounders such as, challenging fetal position, abdominal scarring and uterine fibroid,^67^ fetal position, maternal body habitus and prior uterine surgery,^27^ maternal age, pregnancy duration, birthweight, number of ultrasound examinations,^49^ maternal age and fetal position.^62^

## Discussion

First trimester 3D ultrasonography screening of the brain has the potential for early detection of major abnormalities.^11^ This is supported by the recent recommendation of the ISUOG to perform a 3D, or if not feasible a 2D, neuro-sonographic screening. However, this screening currently relies on manual measurements and visual inspection of the ultrasound scans, which is time-consuming, prone to human errors, and requires additional imaging and interpretation expertise. However, this expertise is not always present in clinical practice.^10^ Computational methods can these analyses, hence the aim of this systematic review was to gain insight into the future research directions needed to bring automated early-pregnancy ultrasound analysis into clinical practice.

In this review the most studied topic was biometry (40%), followed by standard plane detection (29%), segmentation (16%), growth models (7%), visualization (4%), abnormality detection (2%), and quality enhancement (2%). We observed a focus on fully automated learning-based methods, as 76% of the studies used an automatic method and 62% used a learning-based method. However, of the 17 studies using proprietary software available in clinical practice only one is learning-based.^26^ Hence, automated learning-based methods are being developed, but are not yet widely integrated in software used in clinical practice. A possible explanation for this, is that early brain ultrasonography is not yet standard practice worldwide. This is due to the fact that early brain ultrasonography requires a high level of expertise, which is not available in all clinical settings.^10^

The fact that early brain ultrasonography is not yet widely part of clinical practice, is reflected in this review: most studies do not evaluate their method using data from clinical routine practice: the data source is either unclear (15%) or the used data was acquired for research purposes (40%). Moreover, only 13% of the studies used clinical routine data depicting abnormal development in the development of their method, and none of these studies were learning-based. However, abnormal development often leads to structural malformations of the brain, which may be wrongly handled by learning-based methods that are not trained and evaluated for these cases. Hence, our first recommendation is that there should be more focus on developing and evaluating automated learning-based method using clinical routine data depicting both normal and abnormal development. Evaluation on clinical routine data shows the potential benefit a computational method can have in terms of accuracy and time needed, which can lead to integration by commercial parties into already widely used software.

We observed that only 31% of the included studies focused on the first trimester. An explanation might be that there is a limited amount of ultrasound data available for method development, both from clinical practice and research. This is due the fact that recommendation by the ISUOG to perform ultrasonography of the brain in this period is fairly recent and that only two studies shared their data publicly. The dataset by van den Heuvel et al. consists of data covering all three trimesters and can therefore be used to extend methods that were initially developed for the second and third trimester. However, this dataset consists only of 2D slices of the trans-ventricular plane, which are not usable for all studies presented in this review. The second publicly available dataset by Burgos-Artizzu et al. is not covering the first trimester, as it starts at gestational week 18.^23^

Regarding the balance between 2D and 3 ultrasonography, we found 23 (43%) studies using 2D and 31 (57%) studies using 3D ultrasonography. Furthermore, we observed that for the studies using 2D ultrasonography the majority used an automatic (96%), learning-based (65%) method, which was not the case for 3D ultrasonography. This can partly be explained by the two aforementioned publicly available 2D datasets,^23^^,^^44^ and partly by the fact that 3D ultrasonography is not yet widely integrated in clinical practice,^11^ which may lead to fewer available annotations. Hence, the availably of a dataset containing also 3D ultrasound would be beneficial to push the development of automatic, learning-based methods.

Sharing data publicly is in some cases impossible due to privacy regulations; therefore, another good option is to share the program source code. Hence, our second recommendation is that studies should make their dataset and program source code publicly available, especially for 3D ultrasonography during the first trimester. Having the program source code available would lead to more easy comparison between methods, since every research institute can repeat the analysis on their own available data. This was done for none of the studies in the review, but is rapidly becoming the standard as shown in the systematic review of Shen et al., where over the last 10 years the number of open source GitHub repositories, providing code for medical applications, had an annual growth rate of 55%.^79^ Another promising approach is federated learning, where learning-based models are trained locally and only the locally learned models are shared and aggregated.^80^

An additional challenge for 3D ultrasonography is that prior to biometry, growth modelling, abnormality detection and visualization the required standard plane must be detected. Only three (5%) biometry studies in this review detected the appropriate standard plane, all other studies assumed its availability.^25^^,^^30^^,^^47^ However, in clinical practice the appropriate standard planes are not available and have to be found manually by the sonographer. Hence for adoption in clinical practice, the integration of standard plane detection prior to other tasks is a topic that should be studied in more detail.

All studies adequately reported their outcomes qualitatively and/or quantitatively, and additionally 49% of the studies had multiple raters or compared their results to clinical known values. Regarding the evaluation of the learning-based methods, 76% used an external test set and 20% additionally performed cross-validation. Furthermore, for 88% of the studies the annotations were made by, or under supervision of, one or multiple clinical experts.

We found that 16 (29%) studies reported the computational time, which ranged from 70 micro seconds to 25 min. However, when a method is commercialized, optimization steps are taken to minimize the computation time. Furthermore, different computational resources and data-types were used (2D/3D), which also dramatically influences the computational time. Therefore, the computation time reported by each study can not be compared directly, and should be seen as an upper estimate for the possible computation time in clinical practice. For the adoption of such a method in clinical practice, the computation time should be at least be equally fast as manual analysis.

Finally, for the bias assessment of the included studies, we obtained a relatively low median score of 5 out of 9. This is due to the fact that the ErasmusAGE score was initially designed for epidemiological studies, and although it can be adapted, the score is biased towards this type of research. However, currently there is no quality score tailored for computational methods available. Therefore, we have chosen to adapt the ErasmusAGE score, since it is general, well validated and covers key points such as description of methodology and quality of evaluation. As a consequence, in our review, studies scored lower due to the fact that only 5 (9%) of the studies used longitudinal data and 19 (35%) studies did not adjust or analyse for any confounding factors. Although in the evaluation and development of computational method the usage of longitudinal data is not necessary, in some cases, such as growth models, it offers more insight. For the other topics such as biometry, segmentation and standard plane detection analysis of confounding factors is to heavily penalized here. Regarding the analysis of the influence by confounding factors: it is known that image quality of ultrasound varies widely since it is operator and vendor dependent^15^ and is influenced by the BMI of the mother. Another challenging aspect for ultrasound images of the fetus, are the rapid development during early pregnancy and movements during acquisition. Hence, our third recommendation is that every computational method should be evaluated in terms of robustness to at least these key confounders.

We have chosen to focus only on studies involving the brain, since it is clearly visible during early ultrasonography, and its growth and structural development continues throughout pregnancy. However, when abnormal brain development occurs, this may affect the growth and development of the entire embryo and fetus, and thus may also become apparent when monitoring the growth and development of other organs, or the embryo and placenta as a whole. Similarly, other abnormalities not related to the brain, such as spinal and cardiac congenital defects, could affect the development of the brain. The influence of abnormalities in other organs should therefore be taken into account when monitoring growth and development of the brain.

We compared our findings to related systematic reviews, and observed that Fiorentino et al. reviewed deep learning methods for fetal ultrasound of all gestational ages.^16^ They found, in line with our findings, that most studies are about biometry and standard plane detection, and were mainly applied to second and third trimester data. The most studied topic was the cardiac system, followed by the brain. Furthermore, they found three public datasets, two of which we found as well,^23^^,^^44^ and one additional public dataset by Rueda et al. for head and femur segmentation in gestational weeks 21, 28 and 32.^81^ Fiorentino et al. stressed the importance of automated analysis of first trimester data, as it can be used to determine gestational age, whereas biometry at later gestation can only be used to monitor growth progress.

Although less common, during pregnancy MR imaging can be used. We compared our findings to systematic reviews about prenatal and neonatal MR imaging of the brain, and firstly found that segmentation is a well-studied topic, with respectively 33^20^, 16^17^, and 14^18^ automatic methods. Secondly, Oishi et al. found 16 atlases, starting at the 20th week of pregnancy, describing normal growth of the fetal and neonatal brain which are publicly available.^19^ Finally, for the neonatal and infant brain Li et al. found 5 datasets and 7 image processing tools which are publicly available.^17^ Hence, we conclude that prenatal and neonatal MR imaging of the brain is an active field of research and sets a good example for early brain ultrasonography in terms of making both datasets and program source code publicly available. However, similar as for early ultrasonography, prenatal MR imaging of the brain is mainly focused at the second half of pregnancy.

In summary, we recommend the following to improve the adoption of automated learning-based methods in routine clinical practice for early brain ultrasonography:

1. We recommend that in the evaluation of computational methods routine clinical data depicting both normal and abnormal development is used: this will result in a direct reflection of the effect these methods can have in clinical practice.
2. We recommend that studies should make their dataset and program source code publicly available, especially for 3D ultrasonography during the first trimester. Sharing code and/or datasets allows researchers of other institutes to evaluate and extend already existing methods, for example by integration of different tasks such as standard plane detection and biometry.
3. We recommend that studies pay more attention to the influence of the key confounding factors, GA, image quality and body mass index, on the accuracy of their computational methods.

Bringing automatic methods to routine clinical practice will not only drastically reduce the time needed for measurements of the brain and for detection of structural abnormalities, but it will also enable large-scale data-driven model development. These models may provide more detailed insight into the factors, such as lifestyle and epigenetics, that influence growth and development of the fetus. On the one hand, this insight could lead to earlier and better diagnosis of neuro-developmental disorders, which positively influences treatment. On the other hand, this insight could also contribute to prevention of neuro-developmental disorders, for example by introducing periconceptional lifestyle coaching focusing on the factors that influence growth and development.^82^, ^83^, ^84^, ^85^, ^86^, ^87^ Hence, introducing automatic methods to routine clinical practice, especially targeted at early pregnancy, may ultimately lead to better neuro-development of the fetus.

## Contributors

All authors were responsible for the concept and design. MR, SK, RST and WN supervised the project. MR and WB did the study selection and data extraction. AK, MR, SK and WB contributed to data analysis and interpretation. WB wrote the original draft of the manuscript with input from MR. All authors contributed to critical revision of the manuscript, and all authors approved the manuscript. All authors had access to the data presented in this paper and the supplementary material prior to submission.

## Data sharing statement

The search strategy and list of excluded papers during full-text screening is available in the supplementary material; any additional data are available on request.

## Declaration of interests

WN is founder, scientific lead and was stock holder of Quantib BV. Wiro Niessen is board member of the Technical Branch of the Dutch Science Foundation (NWO-TTW). All other authors declare no conflicts of interest.
