# Supplementary material for: Computational methods for the analysis of early-pregnancy brain ultrasonography: a systematic review
Source: eBioMedicine. 2023 Feb 14;89:104466. doi: 10.1016/j.ebiom.2023.104466 (PMC9958260; doi:10.1016/j.ebiom.2023.104466)
Supplement: Supplementary material [file mmc1.docx]

**Supplementary material**

1: Full search terms 2

2: Description ErasmusAGE quality score 4

3: List of studies excluded at full-text screening stage 5

4: Brief explanation of AI terms 8
5: ErasmusAge quality score per item and in total 10

**Supplementary material 1: Full search terms**

**Embase.com (1971-)**

('echography'/exp OR 'echograph'/exp OR ultrasound/de OR 'Doppler flowmetry'/de OR 'three-dimensional imaging'/de OR (echogra* OR echocardiogra* OR ultraso* OR sonogra* OR Doppler* OR ((three-dimension* OR 3-d OR 3d) NEAR/3 imaging)):Ab,ti) AND ('first trimester pregnancy'/de OR 'second trimester pregnancy'/de OR (((first OR 1st OR 1-st OR second OR 2nd OR 2-nd) NEAR/3 (trimester*)) OR ((early) NEAR/3 pregnan*) OR ((6th OR 7th OR 8th OR 9th OR 10th OR 11th OR 12th OR 13th OR 14th OR 15th OR 16th OR 17th OR 18th OR 19th OR 20th OR 6 OR 7 OR 8 OR 9 OR 10 OR 11 OR 12 OR 13 OR 14 OR 15 OR 16 OR 17 OR 18 OR 19 OR 20) NEXT/1 week* NEAR/3 (pregnancy OR gestation*))):Ab,ti) AND ('image analysis'/exp OR 'deep learning'/de OR 'image processing'/de OR 'imaging algorithm'/exp OR 'machine learning'/exp OR 'data processing'/de OR 'information processing'/de OR 'artificial intelligence'/de OR 'computer analysis'/de OR 'computer model'/de OR 'computer prediction'/de OR 'automation'/de OR autoanalysis/de OR 'computer assisted diagnosis'/de OR ((imag* NEAR/3 (analy* OR based* OR processing* OR algorithm* OR technolog*)) OR ((deep OR machine) NEAR/3 learning) OR ((data OR information) NEAR/3 processing) OR (artificial NEAR/3 intelligen*) OR (computer NEAR/3 (analy* OR model* OR predict* OR aided OR assisted OR simulat*)) OR automat* OR autoanaly* OR neural-network OR algorithm*):ab,ti) NOT ([animals]/lim NOT [humans]/lim) AND [English]/lim

**Medline ALL Ovid (1946-)**

(Ultrasonography / OR Ultrasonics / OR Laser-Doppler Flowmetry / OR Ultrasonography, Doppler / OR Imaging, Three-Dimensional / OR (echogra* OR echocardiogra* OR ultraso* OR sonogra* OR Doppler* OR ((three-dimension* OR 3-d OR 3d) ADJ3 imaging)).ab,ti.) AND (Pregnancy Trimester, First / OR Pregnancy Trimester, Second / OR (((first OR 1st OR 1-st OR second OR 2nd OR 2-nd) ADJ3 (trimester*)) OR ((early) ADJ3 pregnan*) OR ((6th OR 7th OR 8th OR 9th OR 10th OR 11th OR 12th OR 13th OR 14th OR 15th OR 16th OR 17th OR 18th OR 19th OR 20th OR 6 OR 7 OR 8 OR 9 OR 10 OR 11 OR 12 OR 13 OR 14 OR 15 OR 16 OR 17 OR 18 OR 19 OR 20) ADJ week ADJ3 (pregnancy OR gestation*))).ab,ti.) AND (Deep Learning/ OR Image Processing, Computer-Assisted/ OR exp Machine Learning/ OR Electronic Data Processing/ OR exp Artificial Intelligence/ OR Computer Simulation/ OR Automation/ OR Autoanalysis/ OR Diagnosis, Computer-Assisted/ OR ((imag* ADJ3 (analy* OR based* OR processing* OR algorithm* OR technolog*)) OR ((deep OR machine) ADJ3 learning) OR ((data OR information) ADJ3 processing) OR (artificial ADJ3 intelligen*) OR (computer ADJ3 (analy* OR model* OR predict* OR aided OR assisted OR simulat*)) OR automat* OR autoanaly* OR neural-network OR algorithm*).ab,ti.) NOT (exp animals/ NOT humans/) AND english.la.

**Web of science Core Collection (1975-)**

TS=(((echogra* OR echocardiogra* OR ultraso* OR sonogra* OR Doppler* OR ((three-dimension* OR 3-d OR 3d) NEAR/2 imaging))) AND ((((first OR 1st OR 1-st OR second OR 2nd OR 2-nd) NEAR/2 (trimester*)) OR ((early) NEAR/2 pregnan*) OR ((6th OR 7th OR 8th OR 9th OR 10th OR 11th OR 12th OR 13th OR 14th OR 15th OR 16th OR 17th OR 18th OR 19th OR 20th OR 6 OR 7 OR 8 OR 9 OR 10 OR 11 OR 12 OR 13 OR 14 OR 15 OR 16 OR 17 OR 18 OR 19 OR 20) NEAR/1 week* NEAR/2 (pregnancy OR gestation*)))) AND (((imag* NEAR/2 (analy* OR based* OR processing* OR algorithm* OR technolog*)) OR ((deep OR machine) NEAR/2 learning) OR ((data OR information) NEAR/2 processing) OR (artificial NEAR/2 intelligen*) OR (computer NEAR/2 (analy* OR model* OR predict* OR aided OR assisted OR simulat*)) OR automat* OR autoanaly* OR neural-network OR algorithm*)))

**Cochrane CENTRAL register of trials (1992-)**

((echogra* OR echocardiogra* OR ultraso* OR sonogra* OR Doppler* OR ((three-dimension* OR "3-d" OR 3d) NEAR/3 imaging)):Ab,ti) AND ((((first OR 1st OR "1-st" OR second OR 2nd OR "2-nd") NEAR/3 (trimester*)) OR ((early) NEAR/3 pregnan*) OR ((6th OR 7th OR 8th OR 9th OR 10th OR 11th OR 12th OR 13th OR 14th OR 15th OR 16th OR 17th OR 18th OR 19th OR 20th OR 6 OR 7 OR 8 OR 9 OR 10 OR 11 OR 12 OR 13 OR 14 OR 15 OR 16 OR 17 OR 18 OR 19 OR 20) NEXT/1 week* NEAR/3 (pregnancy OR gestation*))):Ab,ti) AND (((imag* NEAR/3 (analy* OR based* OR processing* OR algorithm* OR technolog*)) OR ((deep OR machine) NEAR/3 learning) OR ((data OR information) NEAR/3 processing) OR (artificial NEAR/3 intelligen*) OR (computer NEAR/3 (analy* OR model* OR predict* OR aided OR assisted OR simulat*)) OR automat* OR autoanaly* OR neural-network OR algorithm*):ab,ti)

**Google scholar**

echography|echocardiography|ultrasonography|sonogram|Doppler "first|second trimester"|"early pregnancy" "image analysis|based|processing"|algorithm|technology|"deep|machine learning"

**Conference proceedings**We screened title and abstract of full paper conference proceedings, searching for the terms “ultrasound”, “fetus”, “fetal”, “embryo”.

| **Conference** | **Year** |
| --- | --- |
| International Conference on Medical Image Computing and Computer Assisted Intervention (MICCAI) | 2000 - 2021 |
| Workshop on Perinatal, Preterm and Paedicatric Image Analysis, satellite event of MICCAI (PIPPI) | 2016, 2018 - 2021 |
| Workshop on Fetal and InFant Image analysis satellite event of MICCAI (FIFFI) | 2017 |
| IEEE International Symposium on Biomedical Imaging (ISBI) | 2020 - 2021 |
| Information Processing in Medical Imaging (IPMI) | 2001, 2003, 2005, 2007, 2009, 2011, 2013, 2015, 2017, 2019, 2021 |
| Medical Imaging with Deep Learning (MIDL) | 2019, 2020, 2021 |

**Screened systematic reviews**

Torrents-Barrena J, Piella G, Masoller N, Gratacós E, Eixarch E, Ceresa M, Ballester MÁG. Segmentation and classification in MRI and US fetal imaging: Recent trends and future prospects. Med Image Anal. 2019 Jan;51:61-88. doi: 10.1016/j.media.2018.10.003. Epub 2018 Oct 19. PMID: 30390513.

Diniz PHB, Yin Y, Collins S. Deep Learning strategies for Ultrasound in Pregnancy. Eur Med J Reprod Health. 2020 Aug;6(1):73-80. Epub 2020 Aug 25. PMID: 33123376; PMCID: PMC7590498.

**Supplementary material 2: Description ErasmusAGE quality score**

To assess the quality of the studies included in this review the ErasmusAGE quality score was used: a tool composed of five items based on previously published scoring systems that can be adapted to fit the topic of the review. Each of the five items can be allocated either zero, one or two points, giving a total score between zero and ten, with a score of ten point representing the highest quality.

1. **Study design**

- 0: cross-sectional
- 1: longitudinal study
- 2: intervention study

Our review does not include any intervention studies, therefore the highest possible score is 9.

1. **Sample size validation**

- 0: < 35 subjects used for validation of the computational method
- 1: 35 to 250 subjects used for validation of the computational method
- 2: > 250 subjects used for validation of the computational method

The boundaries for the scoring for study size were determined by calculating the first quartile (Q1), median and third quartile (Q3) over the included full-text papers. A score of 0 represents a number of subjects below Q1, a score of 1 represents a number of subjects between Q1 and Q3, and a score of 2 represents a number of subjects higher than Q3.

1. **Description of the computational method**

- 0: results are not reproducible based on description
- 1: key results are reproducible based on description
- 2: all results are reproducible based on description.

1. **Reporting of outcome**

- 0: no appropriate outcome reported, not possible to interpreted the outcome (e.g. only qualitative results without reference values or baseline, inappropriate quantitative metric used)
- 1: qualitative and/or quantitative reported outcome
- 2: qualitative and/or quantitative outcome reported and additionally multiple raters or
  comparison to known clinical outcomes

1. **Influence of confounders**

- 0: not investigated or addressed
- 1: findings are analysed or adjusted for at least one of the key confounding factors (the influence of gestational age, acquisition quality and body mass index).
- 2: findings are analysed or adjusted for at least one of the key confounding factors and additionally, findings are adjusted or analysed for other confounding factors

**Supplementary material 3: List of studies excluded at full-text screening stage**

|  | Author | Year | Title | Exclusion reason |
| --- | --- | --- | --- | --- |
| 1 | Abramowicz | 2021 | Obstetric ultrasound: where are we and where are we going? | No research paper: review article |
| 2 | Adams | 2022 | A telerobotic ultrasound clinic model of ultrasound service delivery to improve access to imaging in rural and remote communities | No US |
| 3 | Arnold | 2009 | 3-D reconstruction of a human fetus with combined holoprosencephaly and cyclopia | GA ≥ 20 weeks |
| 4 | Aftab | 2021 | Simplified models to assess newborn gestational age in low-middle income countries: Findings from a multicountry, prospective cohort study | No US: US imaging not used in computational method |
| 5 | Bastiaansen | 2020 | Atlas-based segmentation of the human embryo using deep learning with minimal supervision | Not about brain |
| 6 | Bano | 2021 | AutoFB: automating fetal biometry estimation from standard ultrasound planes | GA ≥ 20 weeks |
| 7 | Bipin Nair | 2020 | Trimester wise growth prediction of an embryo from ultrasound images | No full text |
| 8 | Caly | 2021 | Machine learning analysis of pregnancy data enables early identification of a subpopulation of newborns with ASD | GA ≥ 20 weeks |
| 9 | Cengiz | 2021 | Automatic fetal gestational age estimation from first trimester scans | Not about brain |
| 10 | Chang | 2000 | Assessment of fetal cerebellar volume using three-dimensional ultrasound | GA ≥ 20 weeks |
| 11 | Chen | 2015 | Automatic fetal ultrasound standard plane detection using knowledge transferred recurrent neural networks | Not about brain |
| 12 | Cheng | 2020 | Unbiased atlas construction for neonatal cortical surfaces via unsupervised learning | GA ≥ 20 weeks: about neonate |
| 13 | Diniz | 2020 | Deep learning strategies for ultrasound in pregnancy | No research paper: review paper |
| 14 | Drukker | 2022 | Function and Safety of SlowflowHD Ultrasound Doppler in Obstetrics | Not about brain |
| 15 | Everwijn | 2019 | Cortical development in fetuses with congenital heart defects using an automated brain-age prediction algorithm | GA ≥ 20 weeks |
| 16 | Exalto | 2016 | 3D ultrasound in early pregnancy | No research paper: conference abstract |
| 17 | Fathima | 2011 | A novel local-phase method of automatic atlas construction in fetal ultrasound | GA ≥ 20 weeks |
| 18 | Fung | 2020 | Achieving accurate estimates of fetal gestational age and personalized predictions of fetal growth based on data from an international prospective cohort study: a population-based machine learning study | GA ≥ 20 weeks |
| 19 | Glonek | 2003 | Prenatal assessment of ventriculomegaly: An anatomical study | No US: no imaging of embryo |
| 20 | Grandjean | 2019 | Optimization of fetal biometry with 3D ultrasound and image recognition (EPICEA): protocol for a prospective cross-sectional study | No research paper: research protocol |
| 21 | Griffiths | 2012 | The use of in utero MRI to supplement ultrasound in the fetus at high risk of developmental brain or spine abnormality | No US: US only used as comparison to MRI |
| 22 | Hata | 2013 | Three- and four-dimensional HDlive rendering image of fetal acrania/exencephaly in early pregnancy | No research paper: case report |
| 23 | Jeba Shiney | 2021 | An improved speckle noise reduction scheme using switching and flagging of noisy data for pre-processing of ultrasonograms in detection of down syndrome during first and second trimesters | Not about brain |
| 24 | Jiao | 2020 | Self-Supervised ultrasound to MRI fetal brain image synthesis | GA ≥ 20 weeks |
| 25 | Kurjak | 1999 | First trimester malformation screening | No research paper: review paper |
| 26 | Kurjak | 2007 | Three-and four-dimensional ultrasonography for the structural and functional evaluation of the fetal face | Not about brain: about the face |
| 27 | Li | 2018 | Standard plane detection in 3D fetal ultrasound using an iterative transformation network | GA ≥ 20 weeks: no GA given |
| 28 | Liu | 2009 | Application of tissue Doppler imaging (TDI) Tei index in the evaluation of fetal ventricular function in the second trimester of pregnancy | Not in English |
| 29 | Matthew | 2022 | Exploring a new paradigm for the fetal anomaly ultrasound scan: Artificial intelligence in real time | GA ≥ 20 weeks |
| 30 | Niel | 2019 | Maturation of the human fetal basioccipital: quantifying shape changes in second and third trimesters using elliptic Fourier analysis | No US |
| 31 | Pavithra | 2015 | Automatic measurement of fetal head structures using ultrasound | No full text |
| 32 | Perez-Gonzalez | 2020 | Probabilistic learning coherent point drift for 3D ultrasound fetal head registration | GA ≥ 20 weeks |
| 33 | Pooh | 2011 | New advances in study of early brain development | No research paper: conference abstract |
| 34 | Pooh | 2011 | Early development of fetal brain structures | No research paper: conference abstract |
| 35 | Pooh | 2017 | Structural studies on fetal brain with silhouette ultrasound | No full text |
| 36 | Rizzo | 2011 | An algorithm based on OmniView technology to reconstruct sagittal and coronal planes of the fetal brain from volume datasets acquired by three-dimensional ultrasound | Duplicate |
| 37 | Rizzo | 2016 | The feasibility of using 5D CNS software in obtaining standard fetal head measurements from volumes acquired by three-dimensional ultrasonography: comparison with two-dimensional ultrasound | Duplicate |
| 38 | Rosen | 2017 | Simulator based obstetric ultrasound training: a prospective, randomized single-blinded study | No US |
| 39 | Rousian | 2010 | Embryonic brain ventricle development and volumetry in virtual reality | No research paper: conference abstract |
| 40 | Selcuk | 2022 | Reference ranges of fetal cisterna magna volume measurements by three-dimensional ultrasonography in the late second trimester considering sonographic experience | GA ≥ 20 weeks |
| 41 | Selvathi | 2022 | Fetal biometric based abnormality detection during prenatal development using deep learning techniques | Not about brain |
| 42 | Stepień | 2008 | The cycloid and skeletonization methods for morphometric analysis of fetal brain vessels | No US |
| 43 | Torrents-Barrena | 2021 | Assessment of radiomics and deep learning for the segmentation of fetal and maternal anatomy in magnetic resonance imaging and ultrasound | No US |
| 44 | Toscano | 2022 | Testing a deep learning algorithm for automatic detection of prenatal ultrasound for under-resourced communities | GA ≥ 20 weeks |
| 45 | Tsai | 2012 | A new automatic algorithm to extract craniofacial measurements from fetal three-dimensional volumes | GA ≥ 20 weeks |
| 46 | Tsai | 2020 | Automatic fetal middle sagittal plane detection in ultrasound using generative adversarial network | Not about brain |
| 47 | Vezzetti | 2014 | Exploiting 3d ultrasound for fetal diagnostic purpose through facial landmarking | Not about brain |
| 48 | Walker | 2022 | Using deep-learning in fetal ultrasound analysis for diagnosis of cystic hygroma in the first trimester | Not about brain |
| 49 | Yan | 2017 | Automatic fetal body and amniotic fluid segmentation from fetal ultrasound images by encoder-decoder network with inner layers | GA ≥ 20 weeks |
| 50 | Yang | 2019 | Towards automated semantic segmentation in prenatal volumetric ultrasound | Not about brain |

Table S1: Studies excluded after full text screening. GA = gestational age, US = ultrasound imaging

**Supplementary material 4: Brief explanation of AI terms**

| Method | Submethod | Abbreviation | Explanation |
| --- | --- | --- | --- |
| Constrained probabilistic tree classifier |  |  | A tree classifier uses a decision tree as a model where every branch represents observations about the image, for example, the presence of a structure, location, or other abstract features. Finally, the image ends up in the endpoints of the branches, called the leaves. The leave in which the image ends up determines the classification results. Probabilistic refers to the fact that the probability for this image to end up in every leave is given. Constrains are applied to the height and number of branches in the tree. |
| Block-matching |  |  | Block-matching is a technique to compare images based on small extracted blocks (also called patches). |
| Data-augmentation |  |  | Data-augmentation means performing (basic) operations on images to create more samples for training of machine learning methods. Often used operations are: flipping, rotation and translation of the images. Typically, the operations are chosen such that the resulting augmented images are biological feasible. |
| Features |  |  |  |
|  | Haar features |  | Haar features compute the sum of the pixel intensities of adjacent (rectangular) regions within the image. Subsequently, the difference between these sums for different regions is calculated and used to categorize subsections within an image. |
|  | Hough transform |  | Hough features are used to detect elliptic and circular shapes. |
| Neural network |  | NN | A neural network is a deep learning model inspired by the structure of the human brain. During training the network takes as input the image, which is passed through several layers. There exist different types of layers, and many neural networks are named after those. Finally, the network outputs the results which are then compared to the ground truth, and using this comparison the network updates the trainable parameters in the layers to improve the result. After training, a neural network directly predicts the results from the input image. |
|  | Dense neural networks |  | A dense neural network is a network that is using only fully connected layers. A fully connected layer consists of neurons and each neuron is connected to each neuron in the previous and in the next layer. |
|  | Convolutional neural network | CNN | A convolutional neural network is a specific type of neural network that uses convolutional operations to alter the image between layers to obtain abstract features. |
|  | Fully convolutional neural network | FCNN | A type of neural network that consists of only convolution-type layers. |

| Method | Submethod | Abbreviation | Explanation |
| --- | --- | --- | --- |
|  | Reinforcement learning |  | Reinforcement learning is a technique that rewards desired updates of the neural network during training and penalizes undesired ones. |
|  | U-net |  | Widely used network architecture for image segmentation proposed by Ronneberger et al. in 2015. |
|  | VGG-net |  | Widely used network architecture for classification, proposed by Simonyan and Zisserman in 2015. |
| Random Forest |  | RF | A Random Forest consists of multiple decision tree classifiers and by combining the result of every tree a more robust classification is typically found. |
|  | Regression Forest |  | A regression forest is similar to the aforementioned Random Forest, but the key difference is that the output is a regression of the data instead of a classification. |
| Support Vector Machine |  | SVM | A support vector machine is a well-known machine learning algorithm, based on finding a decision boundary (called a hyperplane) in an N-dimensional space (with N the number of features) that maximizes the distance between data points of both classes. In a two-dimensional plane this decision boundary would be the best line one can draw between the different classes. |

**Supplementary material 5: ErasmusAge quality score per item and in total**

| Topic | Author | Year | Title | Q1  Study design | Q2  Number of subjects used for validation | Q3  Description of method | Q4  Reporting of outcome | Q5  Influence confounding factors | Total score |
| --- | --- | --- | --- | --- | --- | --- | --- | --- | --- |
| Biometry | Araujo et al. | 2014 | Reference range of fetal cisterna magna volume by three-dimensional ultrasonography using the VOCAL method | 0 | 1 | 2 | 2 | 1 | 6 |
| Biometry | Bertucci et al. | 2011 | Vermian biometric parameters in the normal and abnormal fetal posterior fossa: three-dimensional sonographic study | 0 | 0 | 2 | 1 | 1 | 4 |
| Biometry | Birnbaum et al. | 2021 | Normal cavum veli interpositi at 14–17 gestational weeks: three-dimensional and Doppler transvaginal neurosonographic study | 0 | 1 | 2 | 1 | 1 | 5 |
| Biometry | Budd et al. | 2019 | Confident head circumference measurement from ultrasound with real-time feedback for sonographers | 0 | 2 | 2 | 1 | 0 | 5 |
| Biometry | Carneiro et al. | 2008 | Detection and measurement of fetal anatomies from ultrasound images using a constrained probabilistic boosting tree | 0 | 2 | 2 | 2 | 0 | 6 |
| Biometry | Cinar et al. | 2020 | Reference intervals and reliability of cavum septi pellucidi volume measurements by three-dimensional ultrasound between 19 and 24 weeks' gestation | 0 | 1 | 2 | 2 | 1 | 6 |
| Biometry | Grandjean et al. | 2018 | Artificial intelligence assistance for fetal head biometry: Assessment of automated measurement software | 0 | 0 | 2 | 2 | 1 | 5 |
| Biometry | Hata et al. | 2021 | Transvaginal 3-D power doppler ultrasound evaluation of the fetal brain at 10-13 weeks' gestation | 0 | 1 | 2 | 2 | 1 | 6 |
| Biometry | Pashaj et al. | 2013 | Automated ultrasonographic measurement of basic fetal growth parameters | 0 | 1 | 2 | 1 | 1 | 5 |
| Topic | **Author** | **Year** | **Title** | **Q1**  **Study design** | **Q2**  **Number of subjects used for validation** | **Q3**  **Description of method** | **Q4**  **Reporting of outcome** | **Q5**  **Influence confounding factors** | **Total score** |
| Biometry | Pistorius et al. | 2009 | First trimester neurosonoembryology with automated follicle tracking: Preliminary findings | 0 | 0 | 1 | 1 | 0 | 2 |
| Biometry | Pluym et al. | 2021 | Accuracy of automated three-dimensional ultrasound  imaging technique for fetal head biometry | 0 | 1 | 1 | 2 | 2 | 6 |
| Biometry | Rizzo et al. | 2016 | The feasibility of using 5D CNS software in obtaining standard fetal head measurements from volumes acquired by three-dimensional ultrasonography: Comparison with two-dimensional ultrasound | 0 | 1 | 2 | 2 | 1 | 6 |
| Biometry | Rousian et al. | 2013 | First trimester brain ventricle fluid and embryonic volumes measured by three-dimensional ultrasound with the use of I-Space virtual reality | 1 | 1 | 1 | 2 | 1 | 6 |
| Biometry | Ryou et al. | 2019 | Automated 3D ultrasound biometry planes extraction for first trimester fetal assessment | 0 | 0 | 2 | 2 | 1 | 5 |
| Biometry | Shehzad et al. | 2007 | The correlation between ultrasonic manual and automatic measurements of foetal head and abdominal circumferences | 0 | 1 | 1 | 1 | 0 | 3 |
| Biometry | Sofka et al. | 2014 | Automatic detection and measurement of structures in fetal head ultrasound volumes using sequential estimation and integrated detection network (IDN) | 0 | 1 | 2 | 2 | 1 | 6 |
| Topic | **Author** | **Year** | **Title** | **Q1**  **Study design** | **Q2**  **Number of subjects used for validation** | **Q3**  **Description of method** | **Q4**  **Reporting of outcome** | **Q5**  **Influence confounding factors** | **Total score** |
| Biometry | Van den Heuvel et al. | 2018 | Automated measurement of fetal head circumference using 2D ultrasound images | 0 | 2 | 2 | 2 | 1 | 7 |
| Biometry | Van den Heuvel et al. | 2019 | Automated fetal head detection and circumference estimation from free-hand ultrasound sweeps using deep learning in resource-limited countries | 0 | 1 | 2 | 2 | 1 | 6 |
| Biometry | Verwoerd-Dikkeboom et al. | 2008 | Reliability of three-dimensional sonographic measurements in early pregnancy using virtual reality | 1 | 1 | 2 | 2 | 2 | 8 |
| Biometry | Verwoerd-Dikkeboom et al. | 2010 | Innovative virtual reality measurements for embryonic growth and development | 1 | 0 | 2 | 2 | 1 | 6 |
| Biometry | Yazdi et al. | 2014 | Optimal caliper placement: manual vs automated methods | 0 | 1 | 1 | 2 | 1 | 5 |
| Biometry | Zhang et al. | 2020 | Direct estimation of fetal head circumference from ultrasound images based on regression CNN | 0 | 1 | 2 | 1 | 1 | 5 |
| Standard plane detection | Bastiaansen et al. | 2020 | Towards segmentation and spatial alignment of the human embryonic brain using deep learning for atlas-based registration | 0 | 0 | 2 | 1 | 0 | 3 |
| Standard plane detection | Baumgartner et al. | 2016 | Real-time standard scan plane detection and localisation in fetal ultrasound using fully convolutional neural networks | 0 | 1 | 2 | 1 | 0 | 4 |
| Standard plane detection | Burgos-Artizzu et al. | 2020 | Evaluation of deep convolutional neural networks for automatic classification of common maternal fetal ultrasound planes | 0 | 2 | 2 | 2 | 0 | 6 |
| Standard plane detection | Cuingnet et al. | 2013 | Where is my baby? A fast fetal head auto-alignment in 3D-ultrasound | 0 | 1 | 2 | 1 | 0 | 4 |
| Standard plane detection | Dou et al. | 2021 | Agent with warm start and active termination for plane localization in 3D ultrasound | 0 | 1 | 2 | 1 | 0 | 4 |
| Topic | **Author** | **Year** | **Title** | **Q1**  **Study design** | **Q2**  **Number of subjects used for validation** | **Q3**  **Description of method** | **Q4**  **Reporting of outcome** | **Q5**  **Influence confounding factors** | **Total score** |
| Standard plane detection | Drukker et al. | 2022 | Clinical workflow of sonographers performing fetal anomaly ultrasound scans: deep learning-based analysis | 0 | 2 | 0 | 1 | 2 | 5 |
| Standard plane detection | Kong et al. | 2018 | Automatic and efficient standard plane recognition in fetal ultrasound images via multi-scale dense networks | 0 | 2 | 1 | 1 | 1 | 5 |
| Standard plane detection | Kuklisova-Murgasova et al. | 2013 | Registration of 3D fetal neurosonography and MRI | 0 | 0 | 2 | 2 | 1 | 5 |
| Standard plane detection | Namburete | 2018 | Fully-automated alignment of 3D fetal brain ultrasound to a canonical reference space using multi-task learning | 0 | 1 | 2 | 2 | 1 | 6 |
| Standard plane detection | Rizzo et al. | 2011 | An algorithm based on OmniView technology to reconstruct sagittal and coronal planes of the fetal brain from volume datasets acquired by three-dimensional ultrasound | 0 | 1 | 2 | 2 | 1 | 6 |
| Standard plane detection | Rizzo et al. | 2016 | 5D CNS+ Software for automatically imaging axial, sagittal, and coronal planes of normal and abnormal second-trimester fetal brains | 0 | 1 | 2 | 2 | 0 | 5 |
| Standard plane detection | Sridar et al. | 2016 | Automatic identification of multiple planes of a fetal organ from 2D ultrasound images | 0 | 1 | 1 | 1 | 0 | 3 |
| Standard plane detection | Welp et al. | 2020 | Validation of a semiautomated volumetric approach for fetal neurosonography using 5DCNS+ in clinical data from > 1100 consecutive pregnancies | 0 | 2 | 2 | 1 | 2 | 7 |
| Standard plane detection | Yaqub et al. | 2012 | Automatic detection of local fetal brain structures in ultrasound images | 0 | 1 | 2 | 1 | 0 | 4 |
| Standard plane detection | Yaqub et al. | 2015 | Guided random forests for identification of key fetal anatomy and image categorization in ultrasound scans | 0 | 0 | 1 | 1 | 1 | 3 |
| Topic | **Author** | **Year** | **Title** | **Q1**  **Study design** | **Q2**  **Number of subjects used for validation** | **Q3**  **Description of method** | **Q4**  **Reporting of outcome** | **Q5**  **Influence confounding factors** | **Total score** |
| Standard plane detection | Yeung et al. | 2021 | Learning to map 2D ultrasound images into 3D space with minimal human annotation | 0 | 1 | 2 | 2 | 1 | 6 |
| Segmentation | Al-bander et al. | 2019 | Improving fetal head contour detection by object localisation with deep learning | 0 | 2 | 1 | 1 | 0 | 4 |
| Segmentation | Gofer et al. | 2021 | Machine learning algorithms for classification of first-trimester fetal brain ultrasound images | 0 | 1 | 2 | 1 | 0 | 4 |
| Segmentation | Gutierrez-Becker et al. | 2013 | Automatic segmentation of the fetal cerebellum on ultrasound volumes, using a 3D statistical shape model | 0 | 0 | 2 | 2 | 1 | 5 |
| Segmentation | Hesse et al. | 2022 | Subcortical segmentation of the fetal brain in 3D ultrasound using deep learning | 0 | 2 | 2 | 2 | 1 | 7 |
| Segmentation | Li et al. | 2020 | Automated measurement network for accurate segmentation and parameter modification in fetal head ultrasound images | 0 | 2 | 2 | 1 | 1 | 6 |
| Segmentation | Moccia et al. | 2021 | Mask-R2CNN: a distance-field regression version of Mask-RCNN  for fetal-head delineation in ultrasound images | 0 | 2 | 2 | 1 | 1 | 6 |
| Segmentation | Shu et al. | 2022 | ECAU-Net: Efficient channel attention U-Net for fetal ultrasound cerebellum segmentation | 0 | 1 | 1 | 1 | 0 | 3 |
| Segmentation | Wu et al. | 2017 | Cascaded fully convolutional networks for automatic prenatal ultrasound image segmentation | 0 | 1 | 2 | 1 | 1 | 5 |
| Segmentation | Yaqub et al. | 2013 | Volumetric segmentation of key fetal brain structures in 3D ultrasound | 0 | 0 | 2 | 1 | 0 | 3 |
| Abnormality detection | Zhou et al. | 2021 | Prediction and value of ultrasound image in diagnosis of fetal central nervous system malformation under deep learning algorithm | 0 | 0 | 0 | 1 | 1 | 2 |
| Topic | **Author** | **Year** | **Title** | **Q1**  **Study design** | **Q2**  **Number of subjects used for validation** | **Q3**  **Description of method** | **Q4**  **Reporting of outcome** | **Q5**  **Influence confounding factors** | **Total score** |
| Growth model | Bihoun et al. | 2020 | Fetal biometry assessment with Intergrowth 21st's and Salomon's equations in rural Burkina Faso | 0 | 2 | 1 | 2 | 1 | 6 |
| Growth model | Burgos- Artizzu et al. | 2021 | Analysis of maturation features in fetal brain ultrasound via artificial intelligence for the estimation of gestational age | 1 | 2 | 2 | 2 | 1 | 8 |
| Growth model | Namburete et al. | 2014 | Predicting fetal neurodevelopmental age from ultrasound images | 0 | 0 | 2 | 2 | 0 | 4 |
| Growth model | Wyburd et al. | 2021 | Assessment of regional cortical  development through fissure based gestational age estimation in 3D fetal ultrasound | 0 | 2 | 2 | 1 | 0 | 5 |
| Quality enhancement | Perez-Gonzalez et al. | 2020 | Deep learning spatial compounding from multiple fetal head ultrasound acquisitions | 0 | 0 | 1 | 2 | 1 | 4 |
| Visualization | Pooh et al. | 2016 | Recent advances in 3D ultrasound, silhouette ultrasound, and sonoangiogram in fetal neurology | 0 | 0 | 2 | 1 | 0 | 3 |
| Visualization | Tutschek et al. | 2009 | Virtual reality ultrasound imaging of the normal and abnormal fetal central nervous system | 0 | 0 | 2 | 1 | 1 | 4 |

Table S2. ErasmusAge quality score per item and in total. Q1: study design: cross-sectional (0), longitudinal (1), intervention studies (2). Q2: number of subjects used for validation, the study size: ≤ 35 (0), 35 to 250 (1), ≥ 250 (2). Q3: description of the computational method: not reproducible based on description (0), key results are reproducible based on description (1), all results are reproducible based on description (2). Q4: reporting of the outcome: inadequate (0), qualitative and/or quantitative outcome reported (1), additionally: multiple raters and/or comparison to known clinical outcome (2). Q5: influence of confounding factors: not investigated (0), findings are analysed or adjusted for at least one of the key confounders (the influence of GA, acquisition quality and body mass index) (1), additional analysis or adjustment for confounding factors was performed (2).
